# Asymmetrically Substituted *s*‐Triazine Phosphonates by One‐Step Synthesis

**DOI:** 10.1002/open.202300075

**Published:** 2023-09-19

**Authors:** Claudia Vogt, Carl‐Christoph Höhne, Jennifer Limburger, Alexander König, Tobias Wagener, Edwin Kroke

**Affiliations:** ^1^ TU Bergakademie Freiberg Institut für Anorganische Chemie Leipziger Straße 29 09599 Freiberg Germany; ^2^ Zentrum für effiziente Hochtemoeraturstoffwandlung (ZeHS) Winkler Straße 5 09599 Freiberg Germany; ^3^ Fraunhofer-Institut für Chemische Technologie ICT 76327 Pfinztal Germany; ^4^ BASF Polyurethanes GmbH 49448 Lemförde Germany

**Keywords:** arbuzov, asymmetric, phosphonate, triazine

## Abstract

Asymmetrically substituted *s*‐triazine phosphonates with up to three different phosphonate groups C_3_N_3_RR'R” with R, R’, R”=PO(OR”’) and R”’=for example, methyl, ethyl, isopropyl or *n*‐butyl are interesting as polymer additives like flame retardants. Typically, these compounds are obtained by multiple synthesis steps. However, this leads to high production costs, which are a disadvantage for commercial use. Here we report the one‐step synthesis of mixtures of asymmetrical *s*‐triazine phosphonates which is an easy way to adjust the thermal behaviour and other properties such as viscosities of the compounds. The synthesis is based on a Michaelis–Arbuzov reaction. A complete conversion of the reactants to the target compounds is observed which was proofed by detailed ^1^H, ^13^C and ^31^P NMR investigations and elemental analysis. The thermal behaviour was compared with thermogravimetric analysis (TGA).

## Graphical abstract

Because of restrictions by EACH (European Chemicals Agency), halogen‐based liquid flame retardant materials (FR) are currently under assessment. Therefore, new halogen‐free FR with similar good processing and application properties are of high interest. Thus, a one‐step synthesis of mixtures of *s*‐triazine phosphonates via the Michaelis–Arbuzov reaction is reported.

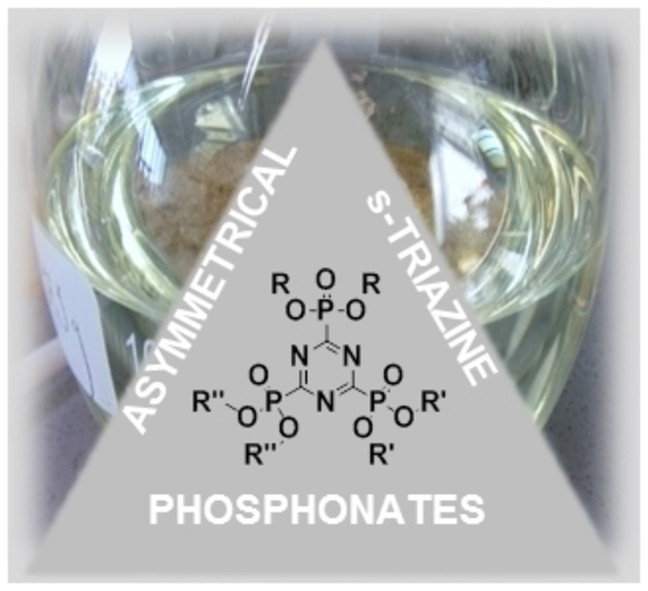

## Introduction

*s*‐Triazines (C_3_N_3_R_3_) are nitrogen‐rich, aromatic heterocyclic structures. By the nucleophilic substitutions of cyanuric chloride (C_3_N_3_Cl_3_) many different compounds are accessible.[^1^, ^2^, ^3^, ^4^] Beside the substitution of all three chlorine atoms with one substituent which leads to symmetric *s*‐triazines (C_3_N_3_R_3_), a gradual nucleophilic substitution^[5]^ with different substituents lead to asymmetrically substituted *s*‐triazines (C_3_N_3_RR'R”). Albericio et al.^[6]^ showed that the substitution depends on the chemical nature of the substituent and the reaction temperature. For example, the typical reactivity of cyanuric chloride with different types of substituents is: alcohol>thiol>amine. This behaviour is used to produce asymmetric *s*‐triazines typically by a step‐by‐step synthesis approach. However, multiple synthesis steps lead to high production costs which is a disadvantage for commercial applications. Manufacturing with as few synthesis and purification steps as possible is to be preferred. We were able to show that asymmetrically substituted *s*‐triazine phosphonates are accessible by a one‐step synthesis.

**Scheme 1:**
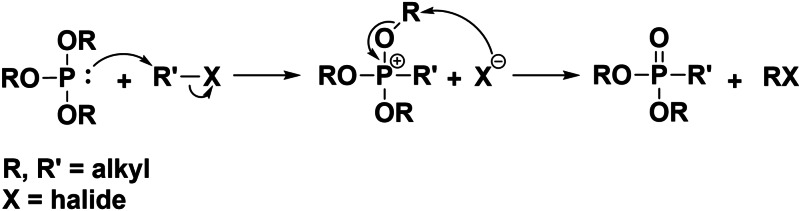
Scheme of an Michealis‐Arbuzov reaction with a trialkyl phosphite and an alkyl halide.

Symmetrical *s*‐triazine phosphonates are known in the literature.[^7^, ^8^, ^9^, ^10^, ^11^] These compounds are obtained by a Michaelis–Arbuzov reaction which is an S_N_2‐reaction developed by Alexander Jerminingeldowitsch Arbuzov and August Michaelis in the 19^th^ century, see Scheme 1.[^12^, ^13^, ^14^, ^15^]

Several different types of symmetrical *s*‐triazine phosphonates are reported until now: Maxim et al. synthesized and crystallized 2,4,6‐tris(dimethoxyphosphonate)‐1,3,5‐triazine and used it as a precursor for trimetal‐complexes.^[16]^ Salmeia et al. obtained 2,4,6‐tris(diethoxyphosphonate)‐1,3,5‐triazine and used it as intermediate to synthesize *N*,*N*’‐bis[4,6‐bis(diethylphosphono)‐1,3,5‐triazin‐yl]‐1,2‐diaminoethane and *N*,*N*’‐bis[4,6‐bis(diethylphosphono)‐1,3,5‐triazin‐yl]‐piperazine. The so‐obtained compounds showed promising flame‐retardant characteristics.^[7]^ Chachlaki et al. also used 2,4,6‐tris(diethoxyphosphonate)‐1,3,5‐triazine to generate the anionic zinc‐[amino(iminio)methyl]phosphonate one‐dimensional coordination polymer.^[17]^ Different research groups used the *s*‐triazine molecule as a precursor and substituted one or two phosphonate groups, for example with amines. The group of Cipolli combined a polyamine with the *s*‐triazine system to get huge networks which leads to a thermoplastic material.^[18]^ Parida et al. used modified *s*‐triazine phosphonate structures (*N*,*N*’‐bis[4,6‐bis(diethylphophono)‐1,3,5‐triazin‐yl]1,2‐diaminoethane) as precursor material for a one‐pot synthesis of mesostructured silica nanoparticles which could be used as a potential drug delivery product or also as a flame retardant.^[19]^ Zuo et al. also published a new synthesis and application of an phosphorus‐containing intumescent flame retardant which is based on an oligomer of poly(2‐morpholinyl‐4‐pentaerythritolphosphate‐1,3,5‐triazine). The oligomer was obtained by a three‐step synthesis with cyanuric chloride as starting material.^[20]^

In the literature, several types of asymmetrical *s*‐triazines were reported.^[1]^ But there are no substances with two or three different phosphonate groups at the *s*‐triazine ring.^[21]^

In this paper, we report the synthesis of mixtures of asymmetric *s*‐triazine phosphonates by a one‐step synthesis using cyanuric chloride and trialkyl phosphite mixtures.

## Results and Discussion

### Synthesis

To obtain pure symmetrically substituted *s*‐triazine phosphonates **TR_3_** as well as mixtures of asymmetrically substituted *s*‐triazine phosphonates **mT/R/R’/R”**, cyanuric chloride and a selected phosphite or mixtures of trialkyl phosphites was brought into reaction. Three different reaction paths were studied, see Table 1. An excess of the phosphite was used to ensure a complete reaction of the cyanuric chloride and to simplify the purification procedures.

**Table 1:**
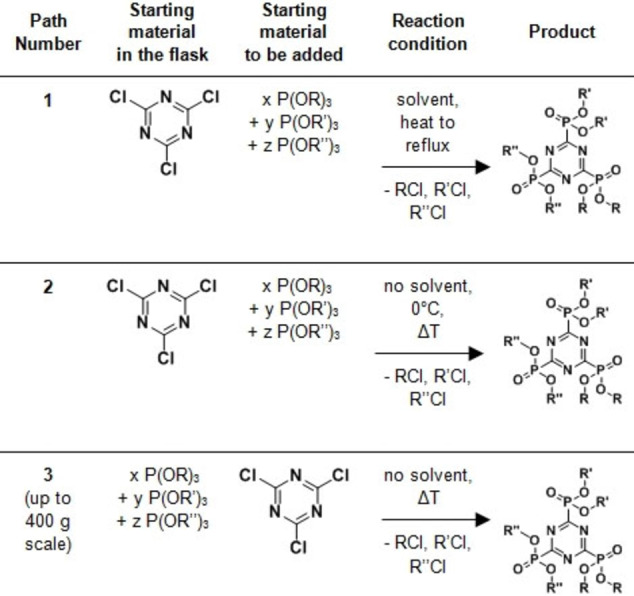
Synthesis routes for symmetric and asymmetric s‐triazine phosphonates with R, R’, R”=alkyl and x, y, z=0, 1, 2, 3.

### Methodology

- Path 1: A liquid mixture of trialkyl phosphites was slowly added to cyanuric chloride dispersed in a solvent.
- Path 2: A liquid mixture of trialkyl phosphites was slowly added to cyanuric chloride powder. No solvent was used.
- Path 3: Cyanuric chloride powder was slowly added to a liquid mixture of trialkyl phosphites. No solvent was used.

The synthesis results for different *s*‐triazine phosphonates and phosphonate mixtures are given in Table 2. No significant differences were observed for the products of the three reaction paths. It does not seem to be essential whether there is an excess of cyanuric chloride (Path 1 and Path 2) or trialkyl phosphite (Path 3) during the reaction. In addition, no solvent is needed during reaction which leads to a simplification of the purification step and important cost savings during production. For a scale up, Path 3 is preferable as the thermal management is easier to handle and due to the fact that the reaction mixtures are liquid during the whole process. All reactions of Path 1 and Path 2 were carried out under inert conditions. Therefore, an oxidation of the alkyl phosphites to alkyl phosphates is not favoured. However, an oxidation to phosphates was also not observed in the reaction of **T**(**nBu**)_**3**_, which was synthesized following Path 3 without strict inert conditions.

**Table 2:** Synthesis results for different *s*‐triazine phosphonates prepared by different reaction paths.

Compound or mixture	Path	Yield [%]	Purity^[a]^ [%]	Aggregate state
2,4,6‐Tris(di‐*n*‐butylphosphonate)‐1,3,5‐triazine **T**(**nBu**)_**3**_	1	quantitative	24	oil
	2	97	95	oil
	3	quantitative	92	oil
2,4,6‐Tris(dimethylphosphonate)‐1,3,5‐triazine **T**(**Me**)_**3**_	1	84	99 (without recrystallization)	solid
	2	23	100	solid
2,4,6‐Tris(diethylphosphonate)‐1,3,5‐triazine **T**(**Et**)_**3**_	1	64	62 (without recrystallization)	solid
2,4,6‐Tris(di‐*i*‐propylphosphonate)‐1,3,5‐triazine **T**(**iPr**)_**3**_	2	94	71 (without recrystallization)	solid
2,4,6‐Tris(di‐*iso‐*decylphosphonate)‐1,3,5‐triazine **T**(**iDec**)_**3**_	2	98	79	oil
**mT/Me/2nBu**	2	99	mixture	oil
**mT/iPr/2nBu**	2	96	mixture	oil
**mT/2iPr/nBu**	2	98	mixture	oil
**mT/Et/iPr/nBu**	2	quantitative	mixture	oil
**mT/Me/Et/nBu**	2	99	mixture	oil
**mT/Me/iPr/nBu**	2	quantitative	mixture	oil

[a] Determined by ^31^P NMR spectroscopy after purification with distillation or recrystallization, synthesis for asymmetrical products leads to mixtures of different substitution patterns.

### Identification of the chemical composition

^13^C NMR and ^31^P NMR spectroscopy were used to study the purity of the *s*‐triazine phosphonates as well as to study the chemical composition of the compounds. ^13^C NMR spectra show the absence of alkyl chlorides and ^31^P NMR spectra show the absence of remaining trialkyl phosphites. All *s*‐triazine phosphonates were obtained with suitable purity, see Table 2.

**Figure 1:**
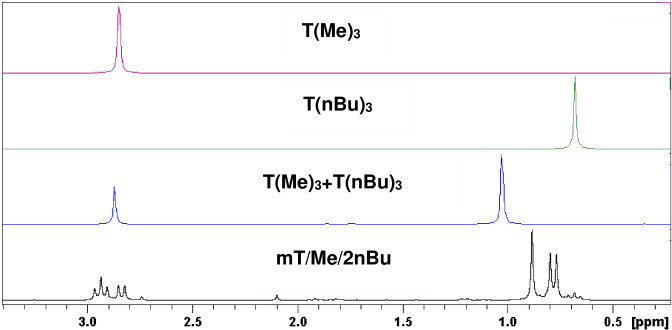
^31^P NMR spectra of **mT/Me/2nBu**, **T**(**Me**)_**3**_ **+T**(**nBu**)_**3**_, **T**(**nBu**)_**3**_, and **T**(**Me**)_**3**_ in CDCl_3_.

In the ^31^P NMR spectrum, only one singlet for the symmetrical triazine phosphonates (see Figure 1) is visible. The different chain length in the **T**(**Me**)_**3**_ and the **T**(**nBu**)_**3**_ at the phosphonates connected to the triazine ring influenced the chemical shift. This leads to a low high‐field shift for longer carbon chains like the *n*‐butyl group in **T**(**nBu**)_**3**_. The chemical shift of **T**(**Me**)_**3**_ is about 2.85 ppm and that of **T**(**nBu**)_**3**_ about 0.68 ppm.

If both symmetrical derivatives are combined in one system **T**(**Me**)_**3**_+**T**(**nBu**)_**3**_, it is possible that a group exchange reaction takes place and more signals will be detected or that both derivatives do not react with each other and no additional signals will be observed. Figure 1 shows the ^31^P NMR spectrum for this experiment: Two singlets are observed. The chemical shift of **T**(**Me**)_**3**_ in the mixture is similar to pure **T**(**Me**)_**3**_. The signal for **T**(**nBu**)_**3**_ in the mixture shows a low downfield shift. No exchange reactions were observed.

**Scheme 2:**
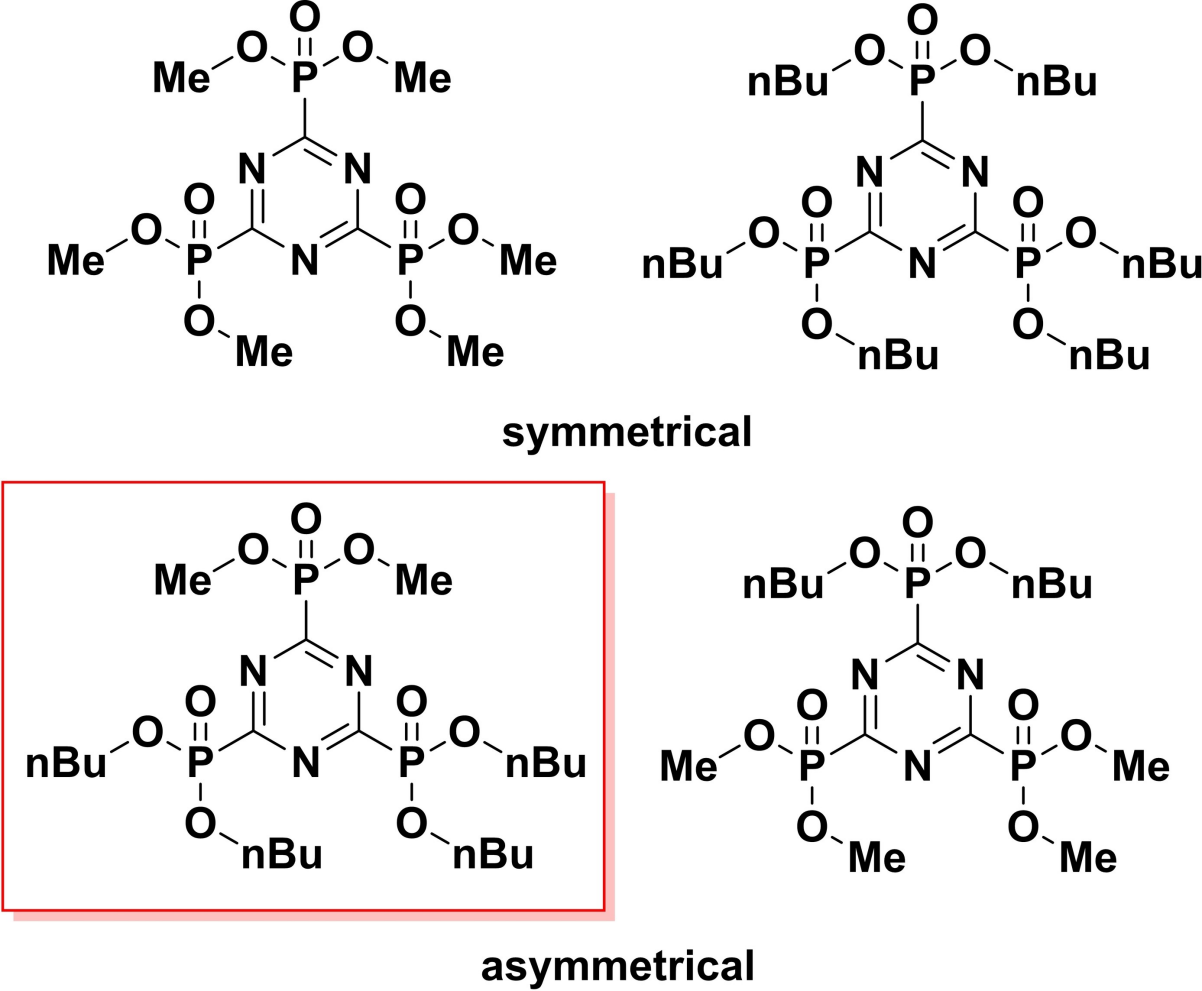
Possible substitution patterns of the reaction mixture of 1 equiv. trimethylphosphite+2 equiv. tri‐*n*‐butylphosphite with 1 equiv. cyanuric chloride=**mT/Me/2nBu**.

A different ^31^P NMR pattern with multiplets is expected for asymmetrically substituted *s*‐triazine phosphonates. For the reaction of a mixture of one eq. of trimethyl phosphite and two eq. of tri‐*n*‐butyl‐phosphite with one eq. of cyanuric chloride, four different *s*‐triazine phosphonates could arise, see Scheme 2. A partial substitution is excluded as a very low chlorine content is typically observed for all of the *s*‐triazine phosphonate synthesis products (see section on chlorine and phosphorous content below).

The ^31^P NMR spectra of a product mixture obtained from a reaction of 1 equiv. C_3_N_3_Cl_3_, 1 equiv. P(OMe)_3_ and 2 equiv. P(OnBu)_3_ denoted **mT/Me/2nBu**, the symmetrical species **T**(**Me**)_**3**_ and **T**(**nBu**)_**3**_ as well as the mixture of them is shown in Figure 1. The mixture shows two signals for the two symmetrical derivatives **T**(**Me**)_**3**_ and **T**(**nBu**)_**3**_, and also some multiplets. The multiplets are caused by intermolecular P−P coupling of the phosphonate groups of the substituted *s*‐triazine ring. An overview about the coupling is given in Table 3.

**Table 3:** Coupling constants ^1^ *J*_(C−P)_, ^3^ *J*_(C−P)_, and ^1^ *J*_(P−P)_ for the different compounds in mixture **mT/Me/2nBu**.

Multiplet ID	^13^C NMR			^31^P NMR		
	Shift [ppm]	^1^ *J*_(C−P)_ [Hz]	^3^ *J*_(C−P)_ [Hz]	Shift [ppm]	^1^ *J*_(P−P)_ [Hz]	Organic group
#1	172.3	259.6	12.5	2.93	4.80	Me, 1
#2	172.2	260.3	12.4	2.84	4.70	Me, 2
#3	171.6	261.3	12.6	0.78	4.82	nBu, 2
#4	171.7	261.1	12.4	0.68	4.69	nBu, 1

**Figure 2:**
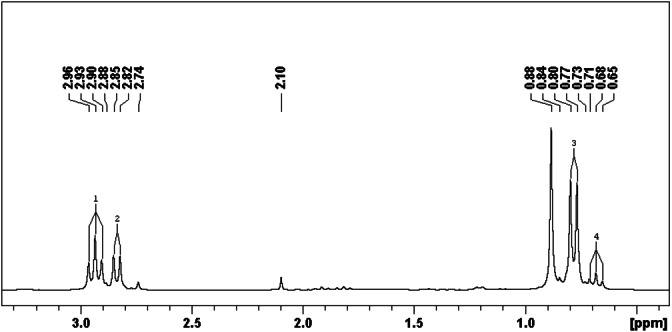
^31^P NMR spectra of **mT/Me/2nBu** in CDCl_3_ with multiplets #1 to #4.

A closer look on the ^31^P NMR spectra of the mixture **mT/Me/2Bu** the symmetrically and asymmetrically substituted products (see Scheme 2) is given in Figure 2. Both multiplets #1 and #3 are caused by **T**(**Me**)(**nBu**)_**2**_ – one phosphonate group substituted with methyl and two phosphonate groups with *n*‐butyl chains (see Scheme 2, red frame). The multiplets #2 and #4 (see Figure 2) are caused by the product which contains one phosphonate group with *n*‐butyl and two phosphonates with methyl groups **T**(**Me**)_**2**_(**nBu**) (see Scheme 2 right side). The additionally detected singlets at 2.74 ppm and 0.88 ppm are caused by the symmetrical products **T**(**Me**)_**3**_ and **T**(**nBu**)_**3**_. The chemical shift of both symmetrical compounds is influenced by the mixture. The signal of **T**(**Me**)_**3**_ shows a high field shift and the signal of **T**(**nBu**)_**3**_ shows a down‐field shift, compared to the ^31^P NMR spectra of the pure symmetrical compounds.

The integrals (see Table 4) of the ^31^P NMR signals were used to estimate the proportion of the *s*‐triazine phosphonates in the mixture, see Table 5.

**Table 4:** Results of the ^31^P NMR spectra of **mT/Me/2nBu** in CDCl_3_.

#		Singlet	Doublet	Triplet
**P**‐**Me**	Chemical shift [ppm]	2.74	2.82	2.93
	Integral area	0.06	0.57	1.00
**P**‐**nBu**	Chemical shift [ppm]	0.88	0.77	0.68
	Integral area	1.40	0.37	1.95

**Table 5:** Quantity [mol %] of the different product species in the reaction mixture obtained from a synthesis using 1 equiv. trimethylphosphite, 2 equiv. tri‐*n*‐butylphosphite and 1 equiv. cyanuric chloride=**mT/Me/2nBu**.

Compound	T(Me)_3_	T(Me)_2_(nBu)	T(nBu)_3_	T(Me)(nBu)_2_
**Quantity by** ^**31**^ **P NMR [%]**	1.1	17.6	26.2	55.1
**Theoretical** **quantity [%]**	3.7	22.2	29.6	44.5

**Scheme 3:**
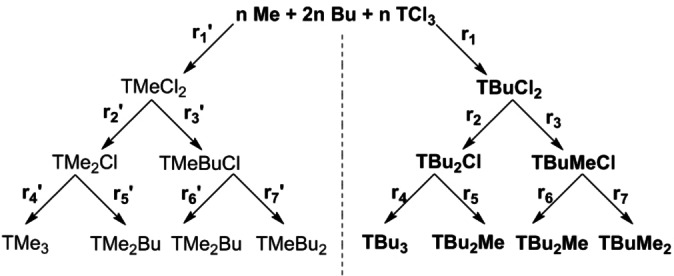
Theoretical reaction paths with r=rate of reaction.

The results of Table 5 are compared with the theoretical quantity of the substances in the product mixture. For this, the reactions rate of each substitution step (see Scheme 3) was assumed to be equal and the concentration of the starting materials was assumed to do not change during the reaction. The excess of tri‐*n*‐butylphosphite in comparison to the trimethylphosphite leads to a ratio of 2/3 to 1/3 considered in the theoretical calculation. Based on these assumptions, the theoretical product yields for the symmetrical compounds are ~23 % for **T**(**nBu**)_**3**_ and ~4 % for **T**(**Me**)_**3**_, ~30 % for **T**(**Me**)_**2**_(**nBu**), and about 45 % for the target product **T**(**Me**)(**nBu**)_**2**_. The experimental quantities of all side products are lower than the theoretical quantities. Only the experimental quantity of the target product **T**(**Me**)(**nBu**)_**2**_ is 10.6 % higher than the theoretical quantity. Accordingly, the target product is formed preferentially and with greater probability than the statistical probability predicts. This is most likely caused by differences of the reaction rates r_1_–r_7_ and r_1_’–r_7_’.

**Figure 3:**
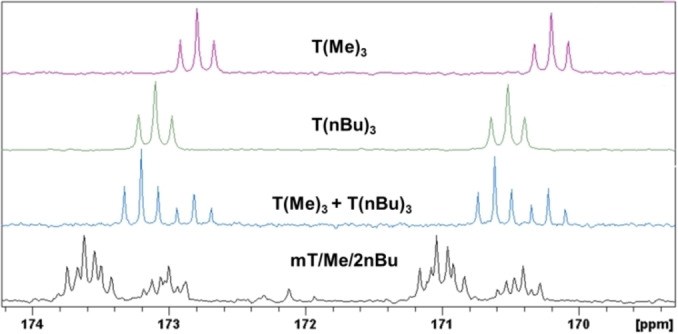
^13^C NMR spectra of **mT/Me/2nBu**, **T**(**Me**)_**3**_ **+T**(**nBu**)_**3**_, **T**(**nBu**)_**3**_, and **T**(**Me**)_**3**_ in CDCl_3_, spectra section of the triazine carbon atom linked to the phosphonate group.

The coupling of the carbon atoms from the *s*‐triazine ring to the phosphorous atoms was used to characterize the chemical composition of the synthesis products via ^13^C NMR spectroscopy. In a symmetrically substituted *s*‐triazine phosphonate, the chemical nature of each phosphonate group is identical which leads to a doublet of triplets in the ^13^C NMR spectrum, with a ^1^ *J* coupling constant for the doublet of about 260 Hz^[7]^ and, for the triplet, a ^3^ *J* coupling constant of about 12 Hz.[^7^, ^22^] Figure 3 shows the double triplet for the symmetrical compounds **T**(**Me**)_**3**_ and **T**(**nBu**)_**3**_. The ^13^C NMR spectrum of a mixture of **T**(**Me**)_**3**_+**T**(**nBu**)_**3**_ is also shown in Figure 3. As discussed on the basis of the ^31^P NMR spectra, no exchange reactions take place. Therefore, two doublets of triplets for the two symmetrical compounds **T**(**Me**)_**3**_ and **T**(**nBu**)_**3**_ appear in the ^13^C NMR spectrum of the mixture. The signal for **T**(**nBu**)_**3**_ shows a low down‐field shift compared to the spectrum obtained for pure **T**(**nBu**)_**3**_.

**Figure 4:**
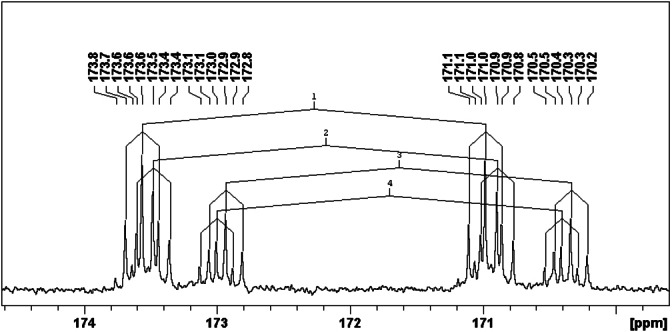
^13^C NMR spectra of **mT/Me/2nBu** in CDCl_3_ with the multiplets #1 to #4, spectra section of the triazine carbon atom linked to the phosphonate group.

The mixture **mT/Me/2nBu** shows additional signals in comparison to the mixture of **T**(**Me**)_**3**_ and **T**(**nBu**)_**3**_ in its ^13^C NMR spectrum (see Figure 4). The signals are in the same range as those for the symmetrical compounds, see Table 3. More multiplets were a result of the additional coupling of the different carbon chains with the P atoms of the phosphonates.

The results of the ^31^P NMR and ^13^C NMR show that the reaction of a phosphite mixture and cyanuric chloride leads to a mixture of symmetrical and asymmetrical *s*‐triazine phosphonates.

### Chlorine and Phosphorus Content

In the Beilstein test^[23]^ of the above mentioned compounds, no green flames were observed, which indicates a very low chlorine content. The formation of chloride by hydrolysis of remaining cyanuric chloride was studied by a self‐made conductivity measuring device for **T**(**nBu**)_**3**_ (triple measurement). The tests indicate a chloride content below 0.5 %. **T**(**nBu**)_**3**_ was additionally checked by elemental analysis. A chlorine content of 0.05±0.01 % was measured.

The phosphorus content was determined with ICP‐OES. For the analysis, the triazine phosphonates were decomposed with a 5 % HNO_3_ solution. The results are shown in Table 6.

**Table 6:** Phosphorus content in [mg L^−1^] measured with ICP‐OES, concentration of the solutions c=5×10^−5^ mol L^−1^.

Compound or mixture	Theoretical P content		Experimental P content		Difference
	[mg L^−1^]	[%]	[mg L^−1^]	[%]	[%]
**T**(**Me**)_**3**_	4.65	22.9	4.20	20.7	9.7
**T**(**Et**)_**3**_	4.65	19.0	3.48	14.2	25.1
**T**(**iPr**)_**3**_	4.65	16.2	4.31	15.0	7.2
**T**(**nBu**)_**3**_ (**Path 2**)	4.65	14.1	4.14	12.6	10.8
**T**(**nBu**)_**3**_ (**Path 3**)	4.65	14.1	4.06	12.4	12.6
**mT/iPr/2nBu**	4.65	14.8	4.15	13.2	10.6
**mT/2iPr/nBu**	4.65	15.4	4.17	13.9	10.3
**mT/Me/2nBu**	4.65	16.2	4.41	15.4	5.1
**mT/Et/iPr/nBu**	4.65	16.2	4.64	16.2	0.2
**mT/Me/Et/iPr**	4.65	19.0	5.38	22.0	15.9
**mT/Me/iPr/nBu**	4.65	17.0	4.53	16.6	2.5

All compounds show deviations of the experimental to the theoretical phosphorus content. **mT/Et/iPr/nBu**, **mT/Me/iPr/nBu**, **mT/Me/2nBu**, **T**(**iPr**)_**3**_ and **T**(**Me**)_**3**_ show the best results with deviations below 10 %. It is assumed that the measured phosphorus content of all molecules is affected by the hydroscopic character of the compounds.

### Influencing the State of Aggregation

The complete substitution of the chlorine atoms of cyanuric chloride with different substituents is often complex and realized by up to three‐step syntheses. Here, we showed that a complete substitution of all chloride atoms by different phosphonate groups is achieved by a one‐step synthesis using a phosphite mixture. The obtained *s*‐triazine phosphonates are classified as halogen‐free products as **T**(**nBu**)_**3**_ synthesized by Path 3 shows a very low remaining chlorine content of 0.05±0.01 %.

The state of aggregation and melting point is important for commercial application of a compound for example as a plastic additive. For plastic additives like flame retardants liquid compounds with low carbon content are of great interest.

The length of the carbon chains of the phosphonate substituents is important to control the state of aggregation of symmetrically substituted *s*‐triazine phosphonates. Phosphonate groups with long carbon chains usually lead to compounds with lower melting point and compounds which are liquid at room temperature. For example, **T**(**Me**)_**3**_, **T**(**Et**)_**3**_ and **T**(**iPr**)_**3**_ are solid at room temperature while **T**(**nBu**)_**3**_ is a liquid^[8]^ with a viscosity similar to olive oil^[24]^ of about 100 mPa ⋅ s at 20 °C.

Asymmetrical substitution and the formation of mixtures is simple method to reduce the melting points with a simultaneous reduction of the overall carbon content, because smaller alkyl substituents lead to higher melting points for symmetrically substituted derivatives. All the studied six mixtures containing asymmetrically substituted *s*‐triazine phosphonates **mT/Me/2nBu**, **mT/iPr/2nBu**, **mT/2iPr/nBu**, **mT/Et/iPr/nBu**, **mT/Me/Et/iPr**, and **mT/Me/iPr/nBu** are hydrophobic viscous liquids at room temperature.

### Thermal Behaviour

**Figure 5:**
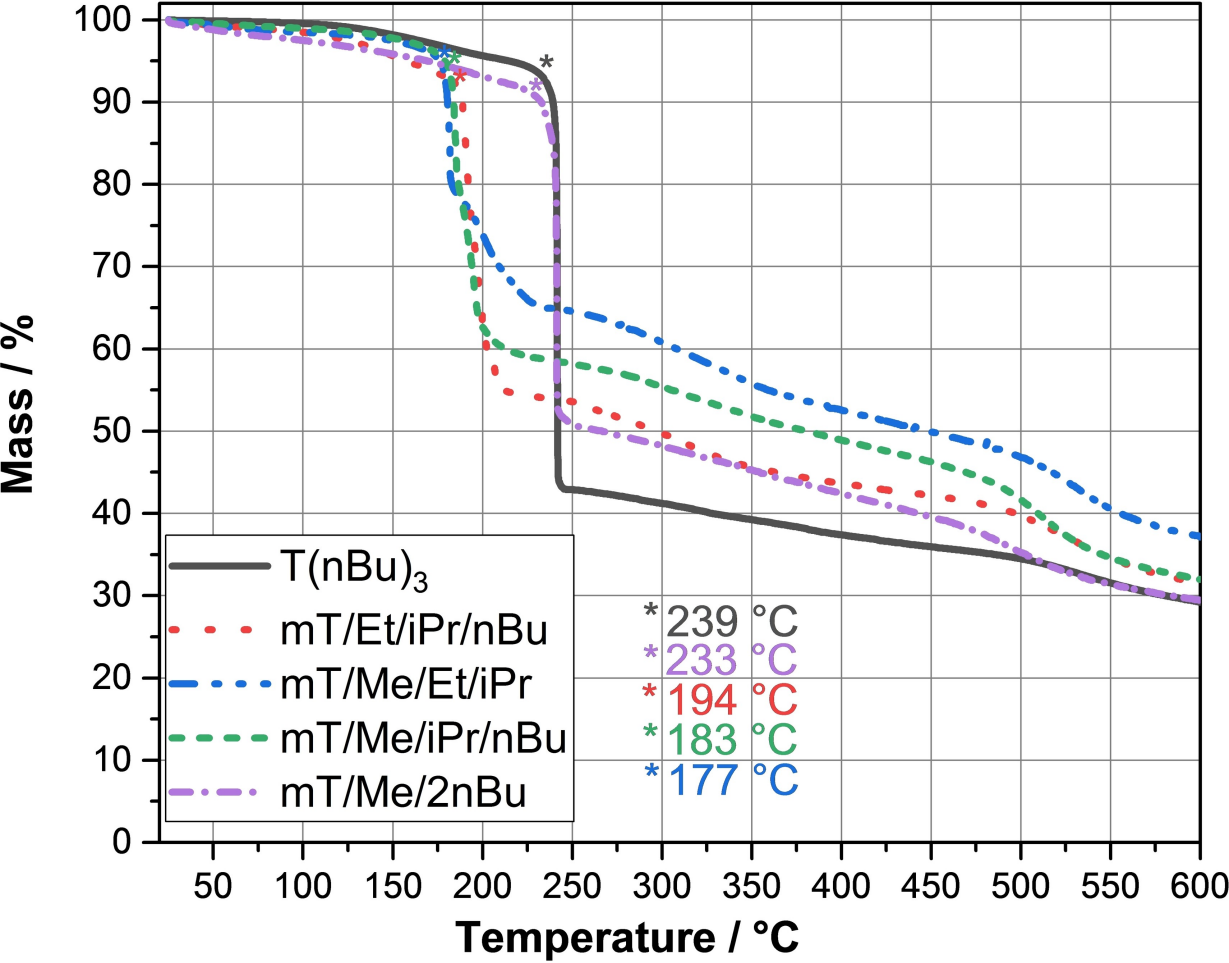
TGA of **T**(**nBu**)_**3**_, **mT/Et/iPr/nBu**, **mT/Me/Et/iPr**, **mT/Me/iPr/nBu**, and **mT/Me/2nBu** (nitrogen atmosphere, 10 K/minute).

Figure 5 and Table 7 show the thermogravimetric analysis (TGA) results for **T**(**nBu**)_**3**_ in comparison with the mixtures containing asymmetrically substituted *s*‐triazine phosphonates **mT/Me/2nBu**, **mT/Et/iPr/nBu**, **mT/Me/Et/iPr**, **mT/Me/2nBu**.

**Table 7:** TGA results of **T**(**nBu**)_**3**_, **mT/Et/iPr/nBu**, **mT/Me/Et/iPr**, **mT/Me/iPr/nBu**, and **mT/Me/2nBu**.

#	T_onset_ [°C]	T_@98 % mass loss_ [°C]	Mass loss at 150–250 °C [%]	Residue at 250 °C [%]
**T**(**nBu**)_**3**_	239.3	214.7	55.3	42.9
**mT/Et/** **iPr/nBu**	194.1	157.1	42.1	54.0
**mT/Me/** **Et/iPr**	177.4	177.1	31.9	65.7
**mT/Me/** **iPr/nBu**	183.4	179.1	39.6	58.1
**mT/Me/** **2nBu**	233.8	169.5	45.1	50.7

**T**(**nBu**)_**3**_ and **mT/Me/2nBu** show high onset temperatures of 239 °C and 233 °C. The other studied specimens show lower onset temperatures of 194 °C, 183 °C, and 177 °C. The higher the amount of *n*‐butyl groups at the phosphonate the higher the resulting decomposition temperature. Methyl groups in **mT/Me/2nBu** lower the onset temperature. This effect can also be transferred to the other samples, resulting in the following order of the decomposition temperature with decreasing chain length of the alkyl groups:

$$T\left(nBu\right){}_{3}>T\left(Me\right)\left(nBu\right){}_{2}>T\left(Et\right)\left(iPr\right)\left(nBu\right)>T(Me)(iPr)(nBu)>T(Me)(Et)(iPr)$$

For example, **T**(**Me**)(**Et**)(**iPr**) shows the lowest chain length and thus the lowest molar mass which leads to the lowest decomposition temperature for the mixture **mT/Me/Et/iPr** of all five studied samples of 177 °C. Thus, the molar masses of the carbon chains at the *s*‐triazine phosphonates affects the resulting decomposition temperature of the molecule and in turn the corresponding mixtures.

The substances are weakly hygroscopic over time if they are stored at air. Therefore, water is released during heating. All studied *s*‐triazine phosphonates show main thermal decomposition steps between 150 °C to 250 °C (see Table 7).

**Figure 6:**
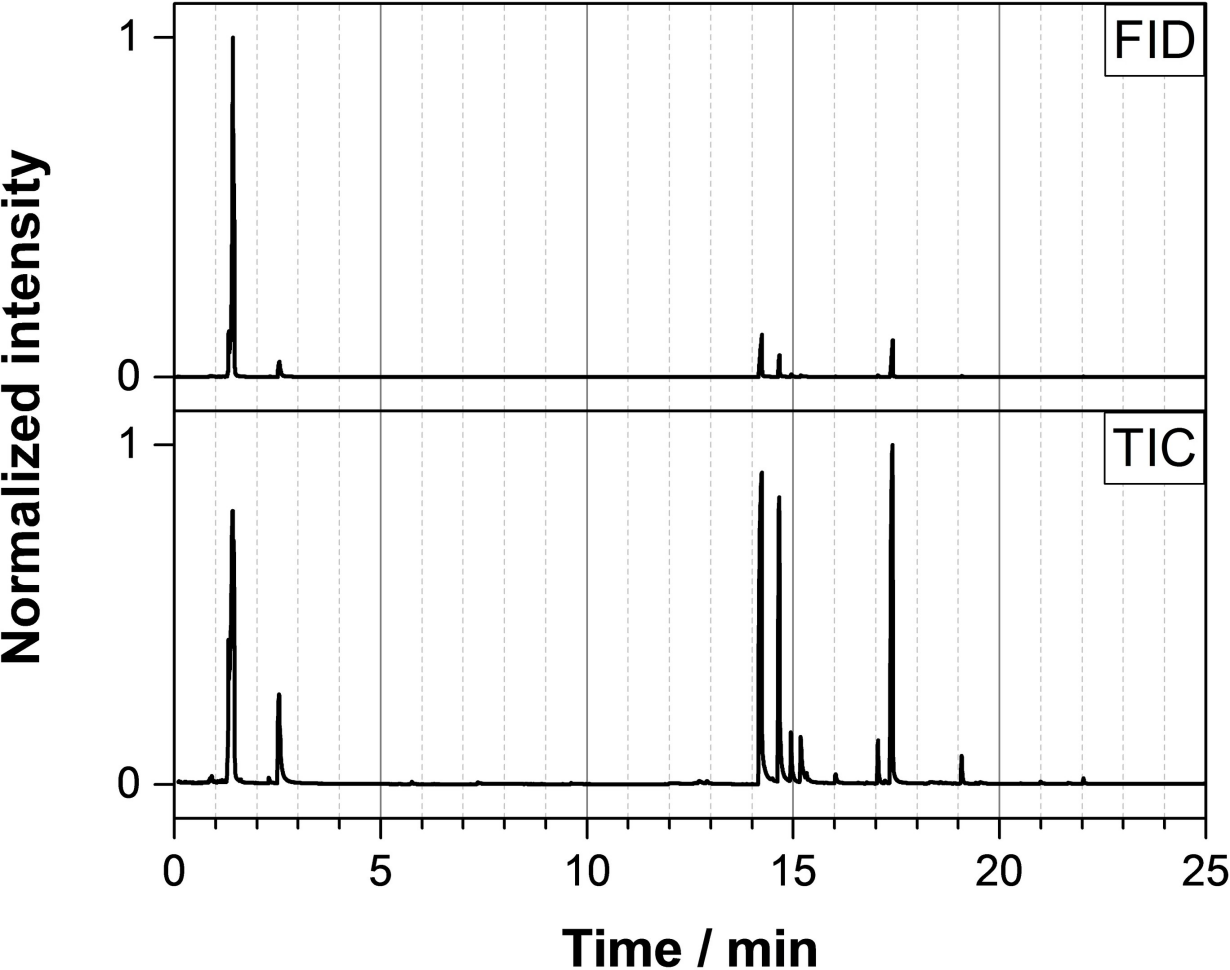
Pyrolysis‐GC‐MS test results of **T**(**nBu**)_**3**_.

The observed mass losses correspond to the theoretical release of “R−O‐R” or the corresponding alkene and alcohol fragments from all three phosphonate groups. **T**(**nBu**)_**3**_ shows a mass loss of 55.3 % which corresponds to a theoretical mass loss of 59.4 % for the thermal release of three dibutyl ether (nBu_2_O) or the corresponding three butene and three butanol fragments from one molecule of **T**(**nBu**)_**3**_. Pyrolysis‐GC‐MS of **T**(**nBu**)_**3**_ performed at 250 °C indicates the release of 2‐butene and 1‐butanol beside the release of different phosphorus‐containing compounds, see Figure 6 and Table 8.

**Table 8:** Main compounds observed by pyrolysis‐GC‐MS of **T**(**nBu**)_**3**_.

Retention time [min]	Compound	Probability [%]	FID	TIC
1.43	2‐Butene	95.9	x	x
2.54	1‐Butanol	92.2	x	x
14.22	Di‐*n*‐butyl hydrogen phosphite	93.4	x	x
14.65	Di‐*n*‐butyl methane phosphonate	95.1	x	x
14.90	Not identified			x
15.18	Not identified			x
17.06	Di‐*n*‐butyl butane phosphonate	91.8		x
17.39	Tri‐*n*‐butyl phosphate	94.9	x	x
19.10	Tri‐*n*‐butyl phosphite	65.7		x

These results suggest the formation of pyro‐ and polyphosphonate *s*‐triazine structures in the condensed phase. However, the observed mass losses are lower than expected for the complete release of all alkoxy groups. The theoretical mass losses are: 53.4 % for **mT/Et/iPr/nBu**, 45.4 % for **mT/Me/Et/iPr**, 51.0 % for **mT/Me/iPr/nBu**, and 53.4 % for **mT/Me/2nBu**. Therefore, the assumed release of dialkyl ethers and corresponding alkenes and alcohols seems to be incomplete.

The thermal activation of the phosphonate groups in the range of 170 °C to 240 °C suggests flame retardant effects in the condensed phase. This aspect will be part of our further investigations.

## Conclusions

Three pathways to synthesize liquid symmetric and asymmetric *s*‐triazine phosphonates were studied. In terms of reaction control, the addition of cyanuric chloride to the phosphite should be chosen.

One‐step synthesis of asymmetric *s*‐triazine phosphonates was performed by using a defined mixture of phosphites. The simplified scheme of the reaction rate combined with the NMR measurements indicated the preferential formation of the target product mixtures containing asymmetrically substituted *s*‐triazines. For example, the asymmetrical product **T**(**Me**)(**nBu**)_**2**_ was obtained with content of about 55 mol % in the mixture **mT/Me/2nBu**. The theoretical quantity was stated to be 45 %.

The thermal behaviour of these *s*‐triazine phosphonates can be adjusted by the length/molecular weight of the organic substituents of the phosphonate groups. Low chain lengths and thus low molecular weights of the organic groups lead to lower decomposition temperatures. Onset temperatures of 177 °C to 239 °C were observed. It is supposed that during the main thermal decomposition step, pyro‐ and polyphosphonate‐bridged *s*‐triazine structures are formed in the condensed phase. Flame retardant investigations are part of ongoing studies.

## Experimental Section

### General

**NMR**: Standard ^1^H, ^13^C and ^31^P NMR spectra were recorded on a Bruker Nanobay 400 NMR spectrometer at 293 K [^1^H (400 MHz), ^13^C (101 MHz), ^31^P (161 MHz)]. The chemical shifts are reported relative to tetramethylsilane.

**ATR**: ATR spectra were recorded at room temperature with a Nicolet 380 FTIR spectrometer in the range 600–4000 cm^−1^

**EA**: The Beilstein test was used for the initial check of the chlorine content. Besides, a self‐made conductivity measuring device for chloride determination was use. It is based on a voltage measurement cell with a silver/silver chloride electrode in a 3 M KCl solution saturated with silver chloride. A defined quantity of the substance was suspended in distilled water and stirred for 3 days at room temperature. For a control measurement, elemental analysis of **T**(**nBu**)_**3**_ was performed extern at a microanalytical laboratory “MIKROLAB Kolbe” (Oberhausen, Germany). For C, N, and H, a CHNS‐analyser Elementar Model Vario Micro Cube was used. Cl was measured by a combustion digestion followed by ion chromatography with an Ionenchromatography Model 883 Plus from Metrohm.

**ICP**‐**OES**: ICP‐OES analyses were recorded on an iCAP 6500 ICP (Thermo Electron) atomic emission spectrometer with ETV‐4000c. The test samples were prepared in a 5 % HNO_3_ solution (5 days at room temperature), which was also measured as a blank value. A calibration was carried out before the measurements. The concentration of the studied solutions were c=5×10^−5^ mol L^−1^.

**TGA**: The TGA measurements were performed with a TG 209 F1 from Netzsch in a nitrogen atmosphere. The heating rate was 10 K/min and the sample mass was about 5 mg.

**Viscosity**: The viscosity measurement was performed with an MCR501 at 20 °C.

**Pyrolysis**‐**GC**‐**MS**: Pyrolysis‐GC‐MS were performed with a Pyrolysis‐GC‐MS from JAS equipped with a pyrolysis oven from Frontier Lab, a GC‐MS 7580 from Agilent and an analytical balance Delta Range XP 26 from Mettler Toledo. The GC was performed with a DB5 column, injection temperature of 250 °C and an oven program from 40 °C to 300 °C with 12 K/min. The pyrolysis was performed from 50 °C to 250 °C with 120 K/min. Released pyrolysis gases were trapped on a cryogenic trap before the GC‐MS analysis was started. Two samples of 1 mg were analysed.

### Synthesis of 2,4,6‐tris(dimethylphosphonate)‐1,3,5‐triazine T(Me)_3_

**Path 1**: 5.05 g (40.7 mmol) trimethylphosphite was slowly added to 2.50 g (13.6 mmol) cyanuric chloride solved in 40 mL benzene with a dropping funnel and stirred under cooling with ice. The dropping funnel was rinsed with additional 10 mL benzene. The mixture was heated to reflux for two hours. Upon reaching room temperature a solid precipitated. This solid was filtered and dried under vacuum. 4.62 g (yield: 84 %) of a white solid were obtained. ^1^H NMR (400 MHz, CDCl_3_): δ=7.30 (CDCl_3_), 4.06 ppm (d, J=12 Hz); ^13^C NMR (100 MHz, CDCl_3_): δ=173.0, 170.5 (J=261 Hz, 12 Hz), 55.3 ppm; ^31^P NMR (161 MHz, CDCl_3_): δ=2.91 ppm; melting point: 115–117 °C.

**Path 2**: 5.05 g (40.7 mmol) trimethyl phosphite was slowly added to 2.50 g (13.6 mmol) cyanuric chloride with a dropping funnel and stirred under cooling with ice. The mixture was heated to reflux for three hours. Upon reaching room temperature a solid precipitated. This solid was filtered and recrystallized with benzene. 1.21 g (yield: 23 %) of a white solid were obtained. ^1^H NMR (400 MHz, CDCl_3_): δ=4.06 ppm (d, J=12 Hz); ^13^C NMR (100 MHz, CDCl_3_): δ=172.8, 170.2 (J=261 Hz, 12 Hz), 55.0 ppm; ^31^P NMR (161 MHz, CDCl_3_): δ=2.85 ppm; melting point: 120 °C; ICP‐OES: P content: 20.7 %.

### Synthesis of 2,4,6‐tris(diethylphosphonate)‐1,3,5‐triazine T(Et)_3_

**Path 1**: 6.76 g (40.7 mmol) triethyl phosphite was slowly added with a dropping funnel to 2.50 g (13.6 mmol) cyanuric chloride suspended in 40 mL benzene and stirred under cooling with ice. The dropping funnel was rinsed with additional 10 mL benzene. The mixture was heated to reflux for two hours. Upon reaching room temperature a solid precipitated. This solid was filtered and dried under vacuum. 4.62 g (yield: 64 %) of a yellow solid were obtained. ^1^H NMR (400 MHz, CDCl_3_): δ=7.26 (CDCl_3_), 4.36 (m), 1.37 (t), 1.27 ppm (m); ^13^C NMR (100 MHz, CDCl_3_): δ=175.0, 173.1, 172.0, 171.0, 128.1 (benzene), 64.9, 16.2 ppm; ^31^P NMR (161 MHz, CDCl_3_): δ=7.19, 0.79 (product), 0.30, −1.10 ppm; melting point: 80 °C.

**Path 2**: 68.93 g (0.41 mol) triethyl phosphite was slowly added to 25.50 g (0.14 mol) cyanuric chloride with a dropping funnel and stirred under cooling with ice. The mixture was heated to reflux for three hours. Upon reaching room temperature a solid precipitated. This solid was filtered and recrystallized with benzene and cyclohexane (1 : 1). 52.76 g (yield: 77 %) of a white solid were obtained. ^1^H NMR (400 MHz, CDCl_3_): δ=7.35 (CDCl_3_), 4.44 (m), 1.45 ppm (t); ^13^C NMR (100 MHz, CDCl_3_): δ=173.5, 170.9, 128.3 (benzene), 65.1, 16.4 ppm; ^31^P NMR (161 MHz, CDCl_3_): δ=0.87 ppm (product); melting point: 95 °C; ICP‐OES: P content: 14.2 %.

### Synthesis of 2,4,6‐tris(di‐i‐propylphosphonate)‐1,3,5‐triazine T(iPr)_3_

**Path 2**: 8.47 g (40.7 mmol) tri‐*i*‐propyl phosphite was slowly added to 2.50 g (13.6 mmol) cyanuric chloride with a dropping funnel and stirred under cooling with ice. The mixture was heated for two hours. Upon reaching room temperature a suspension was obtained. A vacuum distillation was used to remove the impurities. A white solid was obtained and dried under vacuum. 7.34 g (yield: 94 %) of a white solid were obtained. ^1^H NMR (400 MHz, CDCl_3_): δ=7.22 (CDCl_3_), 5.50, 4.56 (m), 0.99 (d), 0.89 ppm (m); ^13^C NMR (100 MHz, CDCl_3_): δ=173.4, 170.8 (J=261 Hz, 12 Hz), 73.4, 71.4, 70.2, 23.8, 23.2 ppm; ^31^P NMR (161 MHz, CDCl_3_): δ=8.80, 8.29, 3.85, −0.87 (product), −1.58, −2.09 ppm; ICP‐OES: P content: 15.0 %.

### Synthesis of 2,4,6‐tris(di‐n‐butylphosphonate)‐1,3,5‐triazine T(nBu)_3_

**Path 1**: 20.36 g (81.3 mmol) tri‐*n*‐butyl phosphite was slowly added to 5.00 g (27.1 mmol) cyanuric chloride solved in 40 mL toluene with a dropping funnel and stirred under cooling with ice. The dropping funnel was rinsed with additional 10 mL toluene. The mixture was heated to reflux for three hours. An oil was obtained at room temperature. A vacuum distillation was used to remove the impurities. 17.8 g (yield: quantitatively) of a yellow oil were obtained. ^1^H NMR (400 MHz, CDCl_3_): δ=7.30 (CDCl_3_), 4.01 (q, OCH_2_), 1.41 (m, CH_2_), 1.10 ppm (m, CH_2_); ^13^C NMR (100 MHz, CDCl_3_): δ=173.0, 170.5 (J=261 Hz, 12 Hz), 68.3, 32.2, 18.3, 13.1 ppm; ^31^P NMR (161 MHz, CDCl_3_): δ=29.74, 7.18, 0.69 ppm (product).

**Path 2**: 145.0 g (0.57 mol) tri‐*n*‐butyl phosphite was slowly added to 35.0 g (0.19 mol) cyanuric chloride with a dropping funnel and stirred under cooling with ice. The mixture was heated for three hours at 100 °C. An oil was obtained at room temperature. A vacuum distillation was used to remove the impurities. 124.1 g of a yellow oil (yield: 97 %) were obtained. Anal calc. C_27_H_54_N_3_O_9_P_3_ (657.66): calc. C 49.31, H 8.28, N 6.39, P 14.13; found: C 49.04±0.01, H 8.26±0.01, N 6.36±0.01, P 14.06±0.01, Cl 0.05±0.01; ^1^H NMR (400 MHz, CDCl_3_): δ=4.01 (q, OCH_2_), 1.41 (m, CH_2_), 1.10 ppm (m, CH_2_); ^13^C NMR (100 MHz, CDCl_3_): δ=173.2, 170.5 (triazine ring), 77.5 (CDCl_3_), 68.1 (OCH_2_), 32.1 (CH_2_), 18.2 (CH_2_), 13.1 ppm (CH_3_); ^31^P NMR (161 MHz, CDCl_3_): δ=29.86, 7.28, 0.68 (product), 0.14, −1.06 ppm; ATR: ν=626 (ν P−O); 809 (P−C); 775–860 (sym‐triazine); 980–1000 (sym‐triazine); 1288 (ν P=O); 1350–1450 (sym‐triazine); 1457 (δ −CH_3_); 1582–1520 (sym‐triazine); 2973 (C−C, −CH_2_/−CH_3_); ICP‐OES: P content: 12.6 %.

**Path 3**: 122.8 g (0.67 mol) cyanuric chloride was slowly added to 501.7 g (2.00 mol) tri‐*n*‐butyl phosphite stirred at 30 °C. The mixture was heated to 90 °C within 4 h and allowed to cool to room temperature overnight. Subsequently, it was heated for another 6 h at 90 °C. An oil was obtained at room temperature. Vacuum distillation was used to remove impurities. 440.1 g of a light‐yellow oil (yield: quantitatively) were obtained. ^1^H NMR (400 MHz, CDCl_3_): δ=7.30 (CDCl_3_), 4.09 (q, OCH_2_), 1.46 (m, CH_2_), 1.15 ppm (m, CH_2_); ^13^C NMR (100 MHz, CDCl_3_): δ=173.1, 170.6 (J=261 Hz, 12 Hz), 68.2, 32.1, 18.4, 13.3 ppm; ^31^P NMR (161 MHz, CDCl_3_): δ=30.07, 10.18, 7.37, 0.74 ppm (product); ICP‐OES: P content: 12.4 %.

### Synthesis of 2,4,6‐tris(di‐iso‐decylphosphonate)‐1,3,5‐triazine T(iDec)_3_

**Path 2**: 40.9 g (81.3 mmol) tri‐*i*‐decylphosphite was slowly added to 5.0 g (27.1 mmol) cyanuric chloride with a dropping funnel and stirred under cooling with ice. The mixture was heated for three hours at 50 °C. An oil was obtained at room temperature. A vacuum distillation was used to remove the impurities. 30.8 g of a yellow oil (yield: 98 %) were obtained. ^1^H NMR (400 MHz, CDCl_3_): δ=7.34 (CDCl_3_), 4.35, 1.78, 1.28, 0.88 ppm; ^13^C NMR (100 MHz, CDCl_3_): δ=173.5, 170.8, 129.6, 125.3, 120.5, 114.3, 68.8, 67.8, 38–10 ppm ^31^P NMR (161 MHz, CDCl_3_): δ=10.37, 7.50, 0.99 (product), 0.45, −3.39 ppm.

### Synthesis of the mixture mT/Me/2nBu

**Path 2**: 31.0 g trimethyl phosphite and 123.0 g tri‐*n*‐butyl phosphite were mixed in a dropping funnel and slowly added to 45.0 g cyanuric chloride and stirred under cooling with ice. The mixture was heated for three hours at 100 °C. An oil was obtained at room temperature. A vacuum distillation was used to remove the impurities. 138.9 g (yield: 99 %) of a yellow oil was obtained. ^1^H NMR (400 MHz, CDCl_3_): δ=4.41 (q, OCH_2_), 4.08 (dd, OCH_3_), 3.80, 3.53, 1.80 (m, CH_2_), 1.50 (m,CH_2_), 0.99 ppm (t, CH_3_); ^13^C NMR (100 MHz, CDCl_3_): δ=173.3, 170.7 (triazine ring), 77.8 (CDCl_3_), 68.7 (OCH_2_), 55.2 (OCH_3_), 32.5 (CH_2_), 18.7 (CH_2_), 13.6 ppm (CH_3_); ^31^P NMR (161 MHz, CDCl_3_): δ=30.07, 10.23, 2.80 (product), 0.75 (product), 0.26, −0.95; ATR: ν=626 (ν P−O); 809 (P−C); 805–820 (triazine); 1110–1160 (triazine); 1288 (ν P=O); 1320–1380 (triazine); 1457 (δ −CH_3_); 1540–1600 (triazine); 2973 (C−C, −CH_2_/−CH_3_); ICP‐OES: P content: 15.4 %.

### Synthesis of the mixture mT/iPr/2nBu

**Path 2**: 108.6 g (0.43 mol) tri‐*n*‐butyl phosphite and 45.2 g (0.22 mol) tri‐*i*‐propyl phosphite were slowly added to 40.0 g (0.22 mol) cyanuric chloride with a dropping funnel and stirred under cooling with ice. The mixture was heated to reflux for three hours. An oil was obtained at room temperature. A vacuum distillation was used to remove the impurities. 129.5 g of a yellow oil (yield: 94 %) were obtained. ^1^H NMR (400 MHz, CDCl_3_): δ=4.71 (m), 4.05 (m), 1.41 (m), 1.14 (m) 0.63 ppm; ^13^C NMR (100 MHz, CDCl_3_): δ=173.7, 173.0, 171.1, 170.5 (J=261 Hz, 12 Hz), 73.7, 68.1, 32.1, 23.9, 23.3, 18.3, 13.2 ppm; ^31^P NMR (161 MHz, CDCl_3_): δ=0.82 (m) (product), 0.69, −0.95 (product); ICP‐OES: P content: 13.2 %.

### Synthesis of the mixture mT/2iPr/nBu

**Path 2**: 20.4 g (81.3 mmol) tri‐*n*‐butyl phosphite and 33.9 g (162.7 mmol) tri‐*i*‐propyl phosphite were slowly added to 15.0 g (81.3 mmol) cyanuric chloride with a dropping funnel and stirred under cooling with ice. The mixture was heated to reflux for three hours. An oil was obtained at room temperature. A vacuum distillation was used to remove the impurities. 43.8 g of a yellow oil (yield: 89 %) were obtained. ^1^H NMR (400 MHz, CDCl_3_): δ=4.94 (m), 4.28 (m), 1.67 (m), 1.36 (m) 0.85 ppm; ^13^C NMR (100 MHz, CDCl_3_): δ=173.3, 172.8, 171.2, 170.8 (J=261 Hz, 12 Hz), 73.8, 68.1, 65.1, 32.1, 24.0, 23.4, 18.3, 13.2 ppm; ^31^P NMR (161 MHz, CDCl_3_): δ=7.48, 0.92 (m) (product), 0.67, −0.75, −0.90 ppm (product); ICP‐OES: P content: 13.9 %.

### Synthesis of the mixture mT/Et/iPr/nBu

**Path 2**: 6.8 g (27.2 mmol) tri‐*n*‐butyl phosphite, 5.6 g (27.2 mmol) tri‐*i*‐propyl phosphite and 4.5 g (27.2 mmol) triethyl phosphite were slowly added to 5.0 g (27.2 mmol) cyanuric chloride with a dropping funnel and stirred under cooling with ice. The mixture was heated for three hours at 100 °C. An oil was obtained at room temperature. A vacuum distillation was used to remove the impurities. 16.5 g of a yellow oil (yield: quantitative) were obtained. ^1^H NMR (400 MHz, CDCl_3_): δ=5.05 (m), 4.45 (m), 4.37 (m), 3.53, 1.77 (m), 1.45 (m), 0.96 ppm; ^13^C NMR (100 MHz, CDCl_3_): δ=173.8, 173.1, 171.1, 170.5 (J=261 Hz, 12 Hz), 73.8, 71.7, 70.5, 68.3, 64.7, 44.4, 39.8, 34.3, 24.0, 19.7, 16.1, 13.1 ppm; ^31^P NMR (161 MHz, CDCl_3_): δ=30.28, 10.16, 7.52, 4.15, 0.88 (m) (product), 0.11, −0.83 ppm (product); ICP‐OES: P content: 16.2 %.

### Synthesis of the mixture mT/Me/Et/iPr

**Path 2**: 56.5 g (271.1 mmol) tri‐*i*‐propyl phosphite, 45.1 g (271.1 mmol) triethyl phosphite and 33.5 g (271.1 mmol) trimethyl phosphite were slowly added to 50.0 g (271.1 mmol) cyanuric chloride with a dropping funnel and stirred under cooling with ice. The mixture was heated to reflux for three hours. An oil was obtained at room temperature. A vacuum distillation was used to remove the impurities. 131.84 g of a yellow oil (yield: 99 %) were obtained. ^1^H NMR (400 MHz, CDCl_3_): δ=4.32 (m), 3.73, 3.35, 3.07, 3.04, 0.74 ppm; ^13^C NMR (100 MHz, CDCl_3_): δ=173.3, 172.7, 172.1, 171.3, 170.6, 170.0 (J=261 Hz, 12 Hz), 73.7, 70.1, 64.4, 54.3, 23.5, 15.9 ppm; ^31^P NMR (161 MHz, CDCl_3_): δ=32.44, 10.22, 6.90, 3.91, 2.66 (product), 1.80, 0.47 (product), −1.06 (m) (product), −1.52 ppm; ICP‐OES: P content: 22.0 %.

### Synthesis of the mixture mT/Me/iPr/nBu

**Path 2**: 3.4 g (13.6 mmol) tri‐*n*‐butyl phosphite, 2.8 g (13.6 mmol) tri‐*i*‐propyl phosphite and 1.7 g (13.6 mmol) trimethyl phosphite were slowly added to 2.5 g (13.6 mmol) cyanuric chloride with a dropping funnel and stirred under cooling with ice. The mixture was heated to reflux for three hours. An oil was obtained at room temperature. A vacuum distillation was used to remove the impurities. 7.6 g of a yellow oil (yield: quantitative) were obtained. ^1^H NMR (400 MHz, CDCl_3_): δ=5.07, 4.25 (m), 3.58, 3.26, 3.11, 2.98, 2.74, 2.53, 0.99, 0.67, 0.16 ppm; ^13^C NMR (100 MHz, CDCl_3_): δ=173.5, 172.8, 172.3, 170.9, 170.3, 169.7 (J=261 Hz, 12 Hz), 73.3, 68.0, 54.6, 44.1, 34.0, 31.9, 23.6, 19.4, 18.1, 12.9 ppm; ^31^P NMR (161 MHz, CDCl_3_): δ=32.22, 29.60, 10.05, 8.62, 7.17, 3.77, 2.60 (product), 1.62, 0.58 (product), −1.10 (m) (product), −3.99 ppm; ICP‐OES: P content: 16.6 %.

## Supporting Information Summary

Additional references have been cited within the Supporting Information.[^25^, ^26^, ^27^, ^28^] The Supporting Information contains the ^1^H, ^13^C and ^31^P NMR spectra of all synthesized compounds and mixtures, viscosity measurement results and the ATR‐IR spectra for the synthesized compounds and mixtures.

## Conflict of interest

The authors declare no conflict of interest.

1

## Supporting information

As a service to our authors and readers, this journal provides supporting information supplied by the authors. Such materials are peer reviewed and may be re‐organized for online delivery, but are not copy‐edited or typeset. Technical support issues arising from supporting information (other than missing files) should be addressed to the authors.

Supporting Information

## Data Availability

The data that support the findings of this study are available from the corresponding author upon reasonable request.
