# Supplementary material for: Asymmetrically Substituted s‐Triazine Phosphonates by One‐Step Synthesis
Source: ChemistryOpen. 2023 Sep 19;12(9):e202300075. doi: 10.1002/open.202300075 (PMC10509391; doi:10.1002/open.202300075)

ChemistryOpen

Supporting Information

Asymmetrically Substituted *s*-Triazine Phosphonates by One-Step Synthesis

Claudia Vogt, Carl-Christoph Höhne,* Jennifer Limburger, Alexander König, Tobias Wagener, and Edwin Kroke*

Contents **Page**

**General**		2
**Syntheses and Literature Data**		2
**References**		2
**Fig. S1**	NMR spectra of 2,4,6-tris(dimethylphosphonate)-1,3,5-triazine **T(Me)$_3$**: a) ^{1}H, b) ^{31}P and c) ^{13}C	3
**Fig. S2**	NMR spectra of 2,4,6-tris(diethylphosphonate)-1,3,5-triazine **T(Et)$_3$**: a) ^{1}H, b) ^{31}P and c) ^{13}C	5
**Fig. S3**	NMR spectra of 2,4,6-tris(di-*i*-propylphosphonate)-1,3,5-triazine **T(iPr)$_3$**: a) ^{1}H, b) ^{31}P and c) ^{13}C	7
**Fig. S4**	NMR spectra of 2,4,6-tris(di-*n*-butylphosphonate)-1,3,5-triazine **T(nBu)$_3$**:a) ^{1}H, b) ^{31}P and c) ^{13}C	9
**Fig. S5**	NMR spectra of 2,4,6-tris(di-*iso*-decylphosphonate)-1,3,5-triazine **T(iDec)$_3$**: a) ^{1}H, b) ^{31}P and c) ^{13}C	11
**Fig. S6**	NMR spectra of the mixture **mT/Me/2nBu**: a) ^{1}H, b) ^{31}P and c) ^{13}C	13
**Fig. S7**	NMR spectra of the mixture **mT/iPr/2nBu**: a) ^{1}H, b) ^{31}P and c) ^{13}C	15
**Fig. S8**	NMR spectra of the mixture **mT/2iPr/nBu**: a) ^{1}H, b) ^{31}P and c) ^{13}C	17
**Fig. S9**	NMR spectra of the mixture **mT/Et/iPr/nBu**: a) ^{1}H, b) ^{31}P and c) ^{13}C	19
**Fig. S10**	NMR spectra of the mixture **mT/Me/Et/iPr**: a) ^{1}H, b) ^{31}P and c) ^{13}C	21
**Fig. S11**	NMR spectra of the mixture **mT/Me/iPr/nBu**: a) ^{1}H, b) ^{31}P and c) ^{13}C	23
**Fig. S12**	Viscosity measurements at 20 °C of 2,4,6-tris(tri-*n*-butylphosphonate)-1,3,5-triazine **T(nBu)$_3$ (B)** and the mixture **mT/Me/2nBu (C)**.	25
**Fig. S13**	ATR-IR spectrum of **T(Me)$_3$**	26
**Fig. S14**	ATR-IR spectrum of **T(Et)$_3$**	27
**Fig. S15**	ATR-IR spectrum of **T(iPr)$_3$**	28
**Fig. S16**	ATR-IR spectrum of **T(nBu)$_3$**	29
**Fig. S17**	ATR-IR spectra of **T(iDec)$_3$**	30
**Fig. S18**	ATR-IR spectrum of the mixture **mT/Me/2nBu**	31
**Fig. S19**	ATR-IR spectrum of the mixture **mT/iPr/2nBu**	32
**Fig. S20**	ATR-IR spectrum of the mixture **mT/2iPr/nBu**	33
**Fig. S21**	ATR-IR spectrum of the mixture **mT/Et/iPr/nBu**	34
**Fig. S22**	ATR-IR spectrum of the mixture **mT/Me/Et/iPr**	35
**Fig. S23**	ATR-IR spectrum of the mixture **mT/Me/iPr/nBu**	36

General

NMR: Standard ^{1}H, ^{13}C and ^{31}P NMR spectra were recorded on a Bruker Nanobay 400 NMR spectrometer at 293 K [^{1}H (400 MHz), ^{13}C (101 MHz), ^{31}P (161 MHz)]. The chemical shifts are reported relative to tetramethylsilane. **ATR**: ATR spectra were recorded at room temperature with a Nicolet 380 FT-IR spectrometer in the range 600-4000 cm^{-1} **EA:** Beilstein test was used for the initial check of the chlorine content. Besides, a self-made conductivity measuring device for chloride determination was use. It is based on a voltage measurement cell with a silver/silverchloride electrode in a 3 M KCl solution saturated with silverchloride. A defined quantity of the substance was suspended in distilled water and stirred for 3 days at room temperature. For a control measurement, elemental analyses were performed extern at a microanalytical laboratory “MIKROLAB Kolbe” (Oberhausen, Germany). For C, N, and H, a CHNS-analyser Elementar Model Vario Micro Cube was used. Cl was measured by a combustion digestion with an Ionenchromatography Model 883 Plus from Metrohm. **ICP-OES:** ICP-OES analyses were recorded on an iCAP 6500 ICP (Thermo Electron) atomic emission spectrometer with ETV-4000c. The test samples were prepared in a 5 % HNO_3 solution (5 days at room temperature), which was also measured as a blank value. A calibration was carried out before the measurements. The concentration of the studied solutions were $c = 5\cdot10^{-5}$ $mol\cdot L^{-1}$. **TGA:** The TGA measurements were performed with a TG 209 F1 from Netzsch in a nitrogen atmosphere. The heating rate was 10 K/min and the sample mass was about 5 mg. **Viscosity:** The viscosity measurement was performed with an MCR501 at 20 °C. **Pyrolysis-GC-MS:** Pyrolysis-GC-MS were performed with a Pyrolysis-GC-MS from JAS equipped with a pyrolysis oven from Frontier Lab, a GC–MS 7580 from Agilent and an analytical balance Delta Range XP 26 from Mettler Toledo. The GC was performed with a DB5 column, injection temperature of 250 °C and an oven program from 40 °C to 300 °C with 12 K/min. The pyrolysis was performed from 50 °C to 250 °C with 120 K/min. Released pyrolysis gases were trapped on a cryogenic trap before the GC-MS analysis was started. Two samples of 1 mg were analysed. **ATR-IR:** The ATR-IR spectra were detected on a Nicolet 380 FT-IR Spectrometer with 128 scans and a resolution of 4.

Syntheses and Literature Data

Some general synthesis procedures and few experimental data (mainly elemental analyses) for the compounds 2,4,6-tris(dimethylphosphonate)-1,3,5-triazine **T(Me)$_3$**, 2,4,6-tris(diethylphosphonate)-1,3,5-triazine **T(Et)$_3$**, 2,4,6-tris(di-*i*-propylphosphonate)-1,3,5-triazine **T(iPr)$_3$**, and 2,4,6-tris(di-*n*-butylphosphonate)-1,3,5-triazine **T(nBu)$_3$**:are mentioned in the literature, namely in the CAS Registry Data Base, as well as in references [1], [8], [11] and [16] of the main part of this paper. Besides, the four compounds have been mentioned in the patent literature, **T(Me)$_3$** and **T(Et)$_3$** in [25,26], **T(iPr)$_3$** in [27] and **T(nBu)$_3$** in [25,28].

References

[1] G. Blotny, *Tetrahedron* **2006**, *62*, 9507–9522.

[8] W. Hewertson, R. A. Shaw, B. C. Smith, *J. Chem. Soc.* **1963**, 1670-1675.

[11] S. von Angerer, *Science of Synthesis* **2004***,* 17, 449-583.

[16] C. Maxim, A. Matni, M. Geoffroy, M. Andruh, N. G. R. Hearns, R. Clérac, N. Avarvari, *New J. Chem.* **2010**, *34*, 2319-2327.

[25] G. F. D’Alelio, US 3158450, **1964**-11-24.

[26] R. Cipolli, C. Rossi, R. Oriani, E. Masarati, G. Nucida, EP 547714 A1, **1993**-06-23.

[27] E. Bencini, G. C. Fasulo, G. Goffredi, F. Mori, IT 93MI2496 A1, **1995**-05-27.

[28] J. S. Leake, WO 9312173 A2, **1993**-06-24.

Figure S1: NMR spectra of 2,4,6-tris(dimethylphosphonate)-1,3,5-triazine T(Me)$_3$: a) ^{1}H, b) ^{31}P and c) ^{13}C

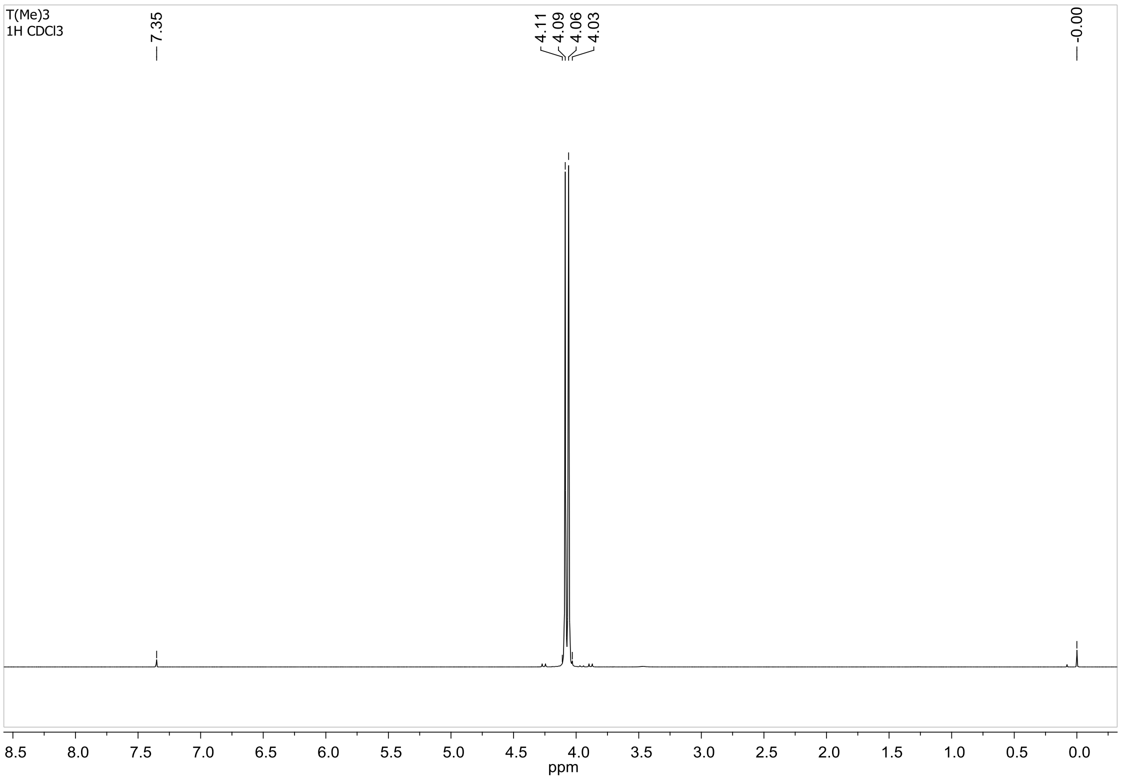

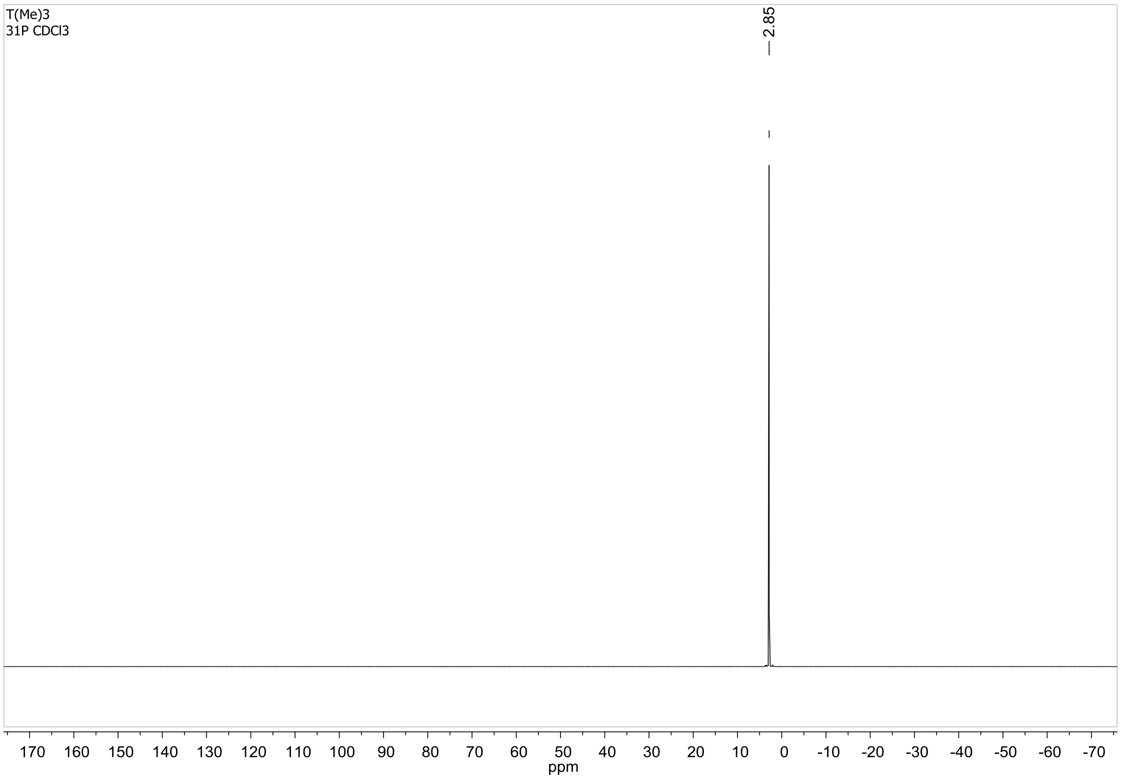

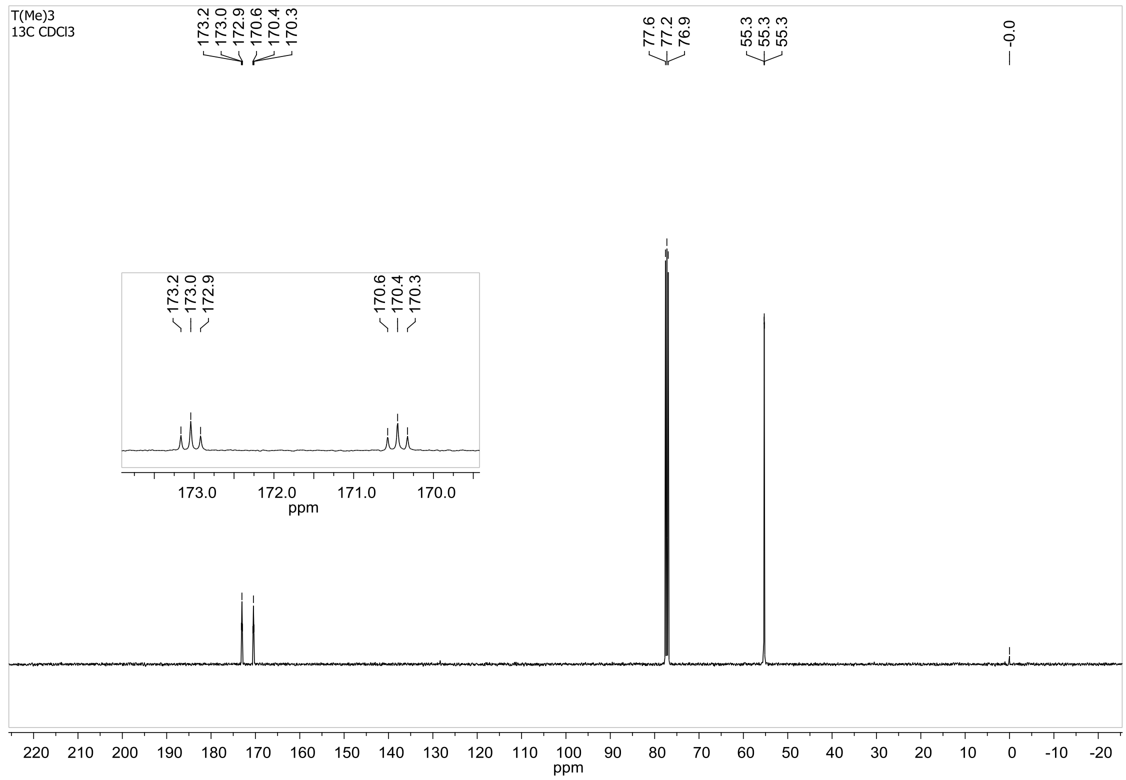
T(Me)3
13C CDCl3
173.2
173.0
172.9
170.6
170.4
170.3
77.6
77.2
76.9
55.3
55.3
55.3
-0.0
173.0
172.0
171.0
170.0
ppm
220
210
200
190
180
170
160
150
140
130
120
110
100
90
80
70
60
50
40
30
20
10
0
-10
-20
ppm

Figure S2: NMR spectra of 2,4,6-tris(diethylphosphonate)-1,3,5-triazine $T(Et)_3$: a) ^{1}H, b) ^{31}P and c) ^{13}C

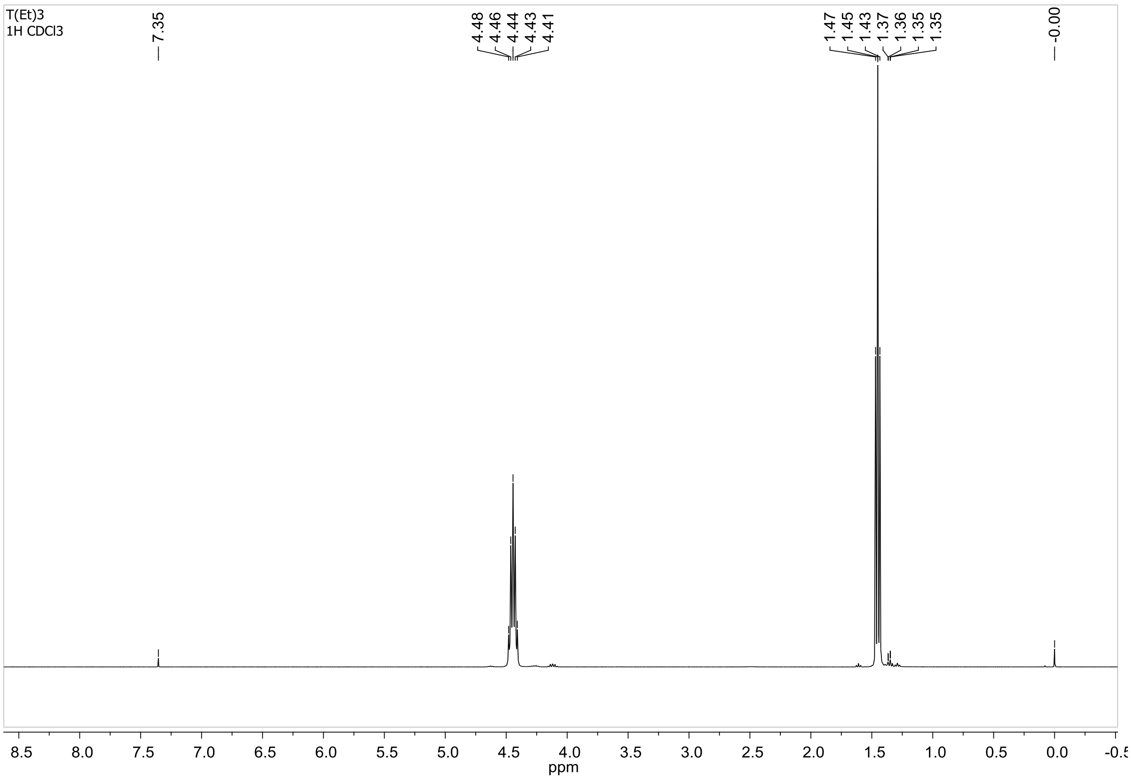

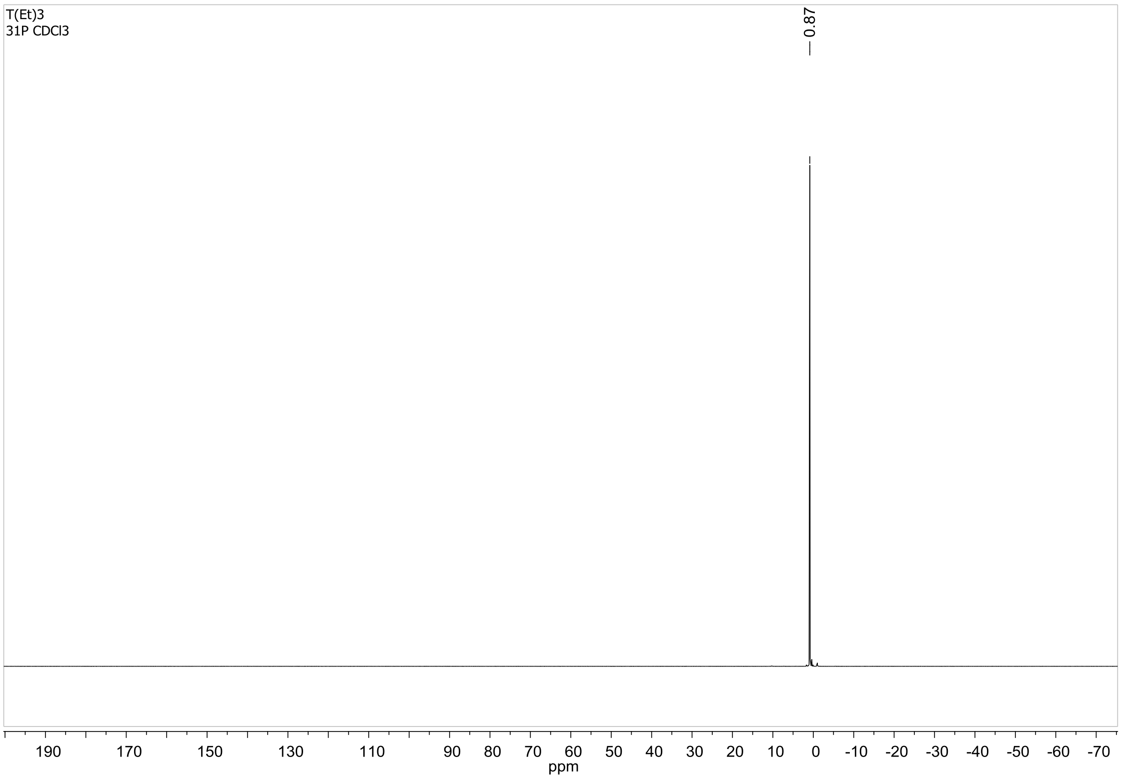

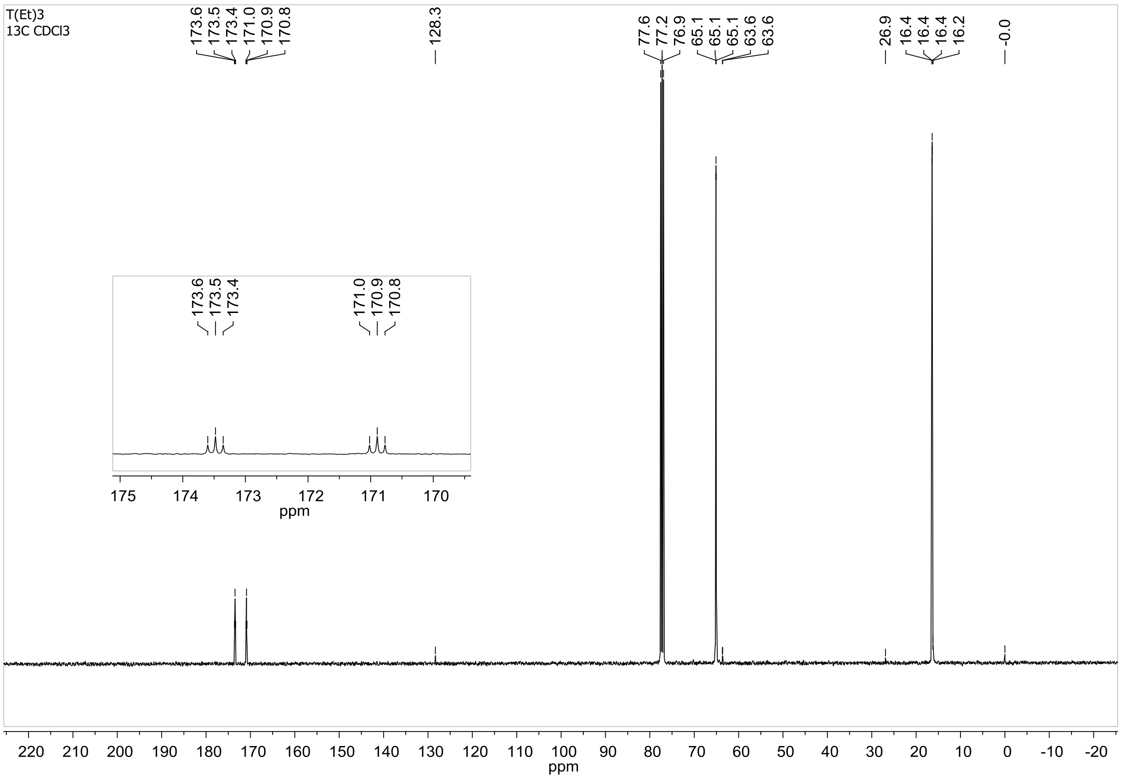
T(Et)3
13C CDCl3
173.6
173.5
173.4
171.0
170.9
170.8
128.3
77.6
77.2
76.9
65.1
65.1
65.1
63.6
63.6
26.9
16.4
16.4
16.4
16.2
-0.0
175
174
173
172
171
170
ppm
220
210
200
190
180
170
160
150
140
130
120
110
100
90
80
70
60
50
40
30
20
10
0
-10
-20
ppm

Figure S3: NMR spectra of 2,4,6-tris(di-*i*-propylphosphonate)-1,3,5-triazine T(iPr)$_3$: a) ^{1}H, b) ^{31}P and c) ^{13}C

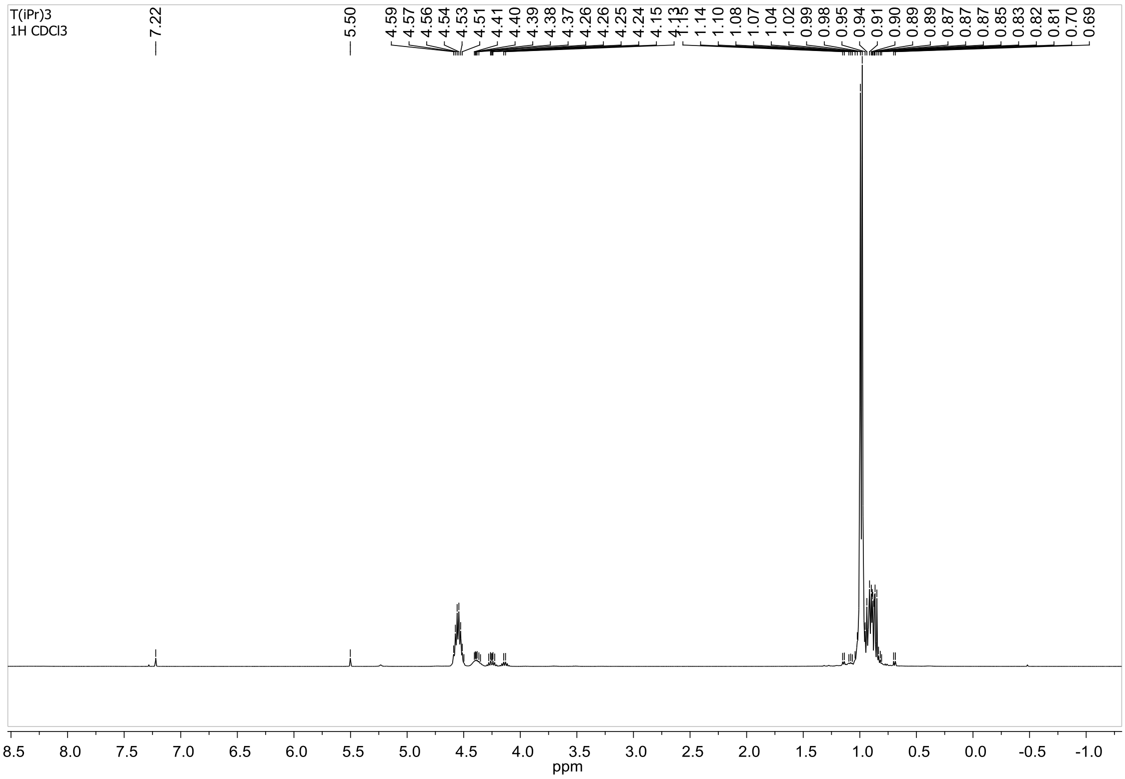

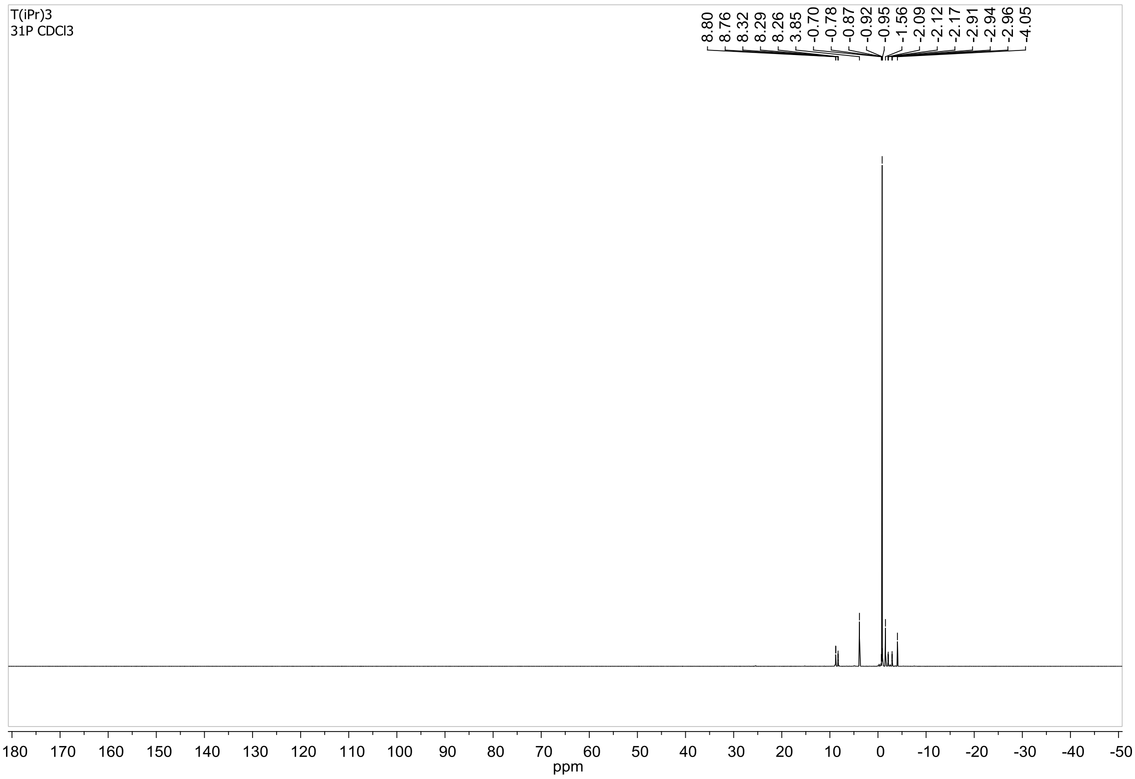
T(iPr)3
31P CDCl3
8.80
8.76
8.32
8.29
8.26
3.85
-0.70
-0.78
-0.87
-0.92
-0.95
-1.56
-2.09
-2.12
-2.17
-2.91
-2.94
-2.96
-4.05
180 170 160 150 140 130 120 110 100 90 80 70 60 50 40 30 20 10 0 -10 -20 -30 -40 -50
ppm

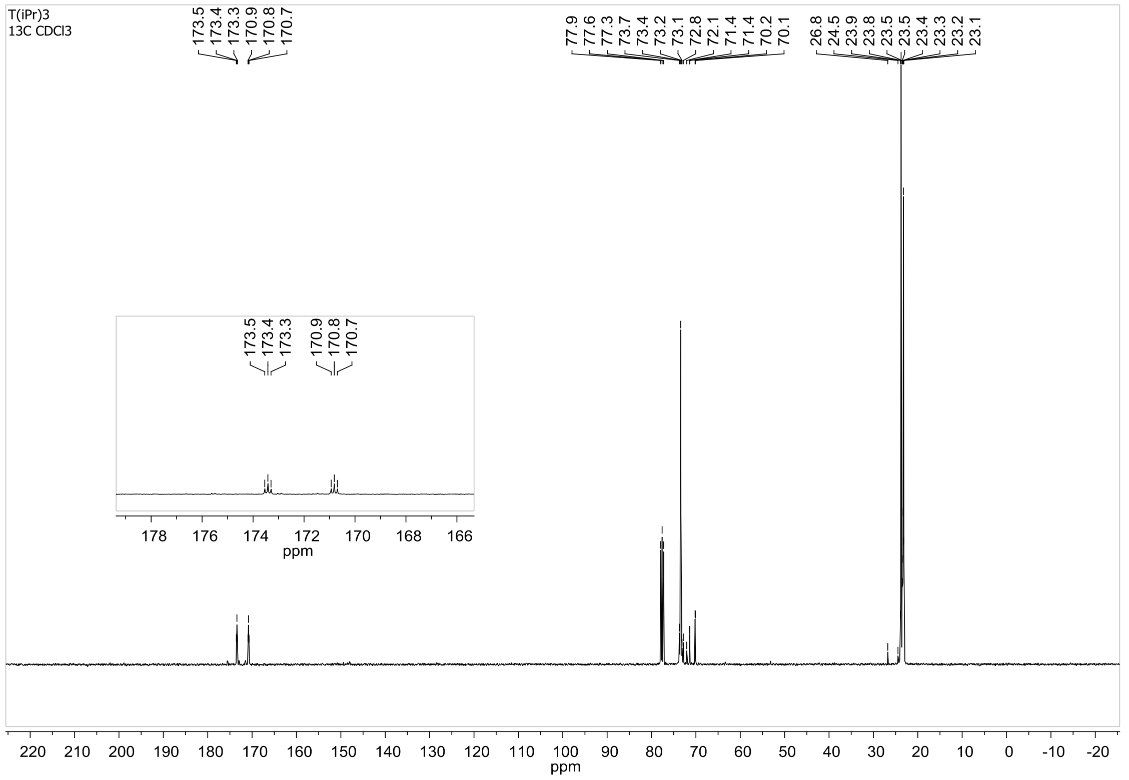
T(iPr)3
13C CDCl3
173.5
173.4
173.3
170.9
170.8
170.7
77.9
77.6
77.3
73.7
73.4
73.2
73.1
72.8
72.1
71.4
71.4
70.2
70.1
26.8
24.5
23.9
23.8
23.5
23.5
23.4
23.3
23.2
23.1
173.5
173.4
173.3
170.9
170.8
170.7
178 176 174 172 170 168 166
ppm
220 210 200 190 180 170 160 150 140 130 120 110 100 90 80 70 60 50 40 30 20 10 0 -10 -20
ppm

Figure S4: NMR spectra of 2,4,6-tris(di-*n*-butylphosphonate)-1,3,5-triazine T(nBu)$_3$: a) ^{1}H, b) ^{31}P and c) ^{13}C

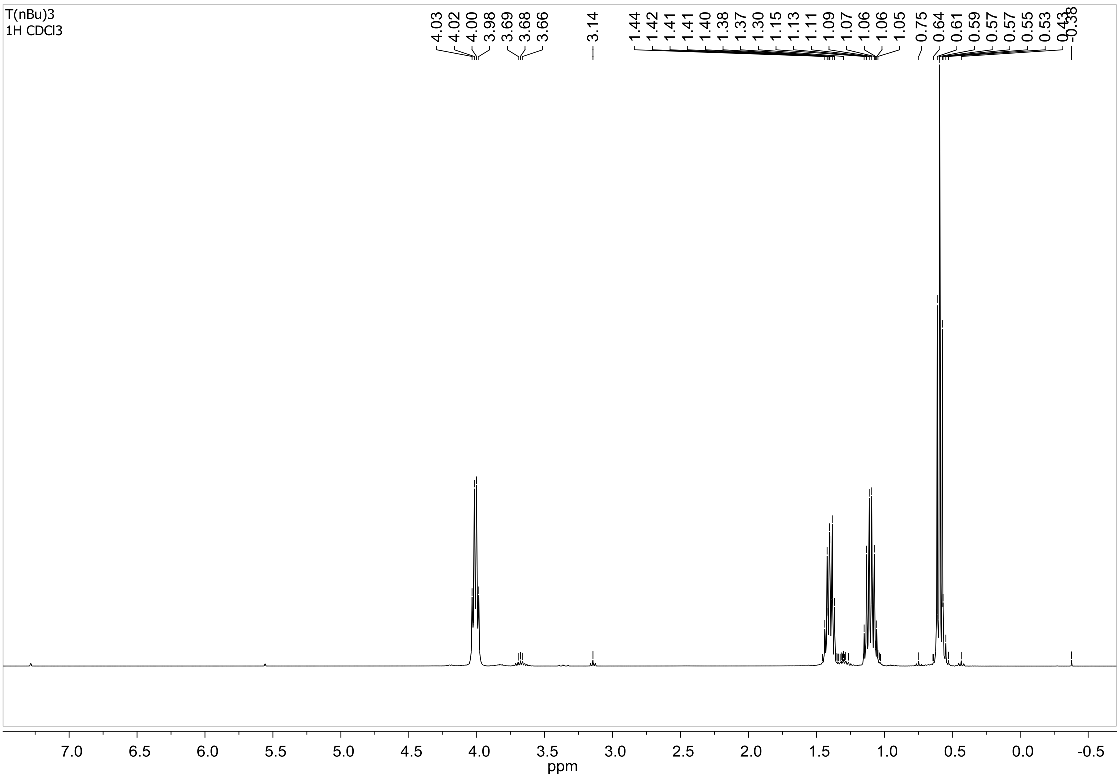

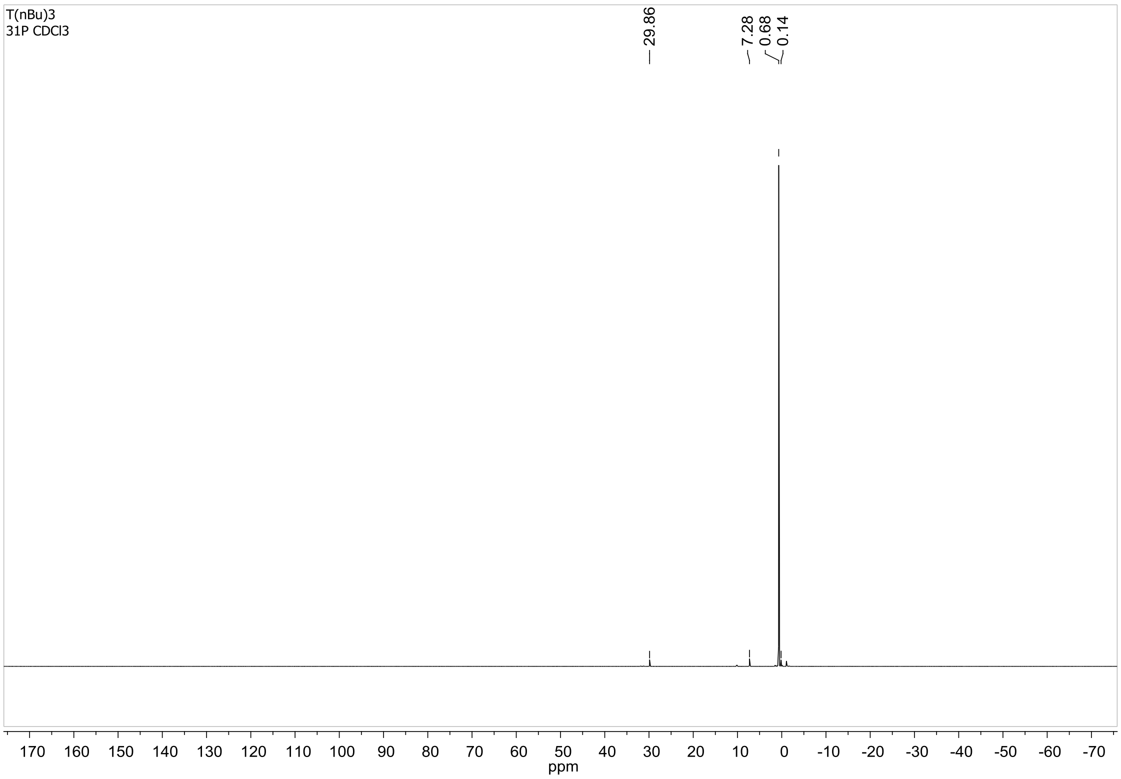

T(nBu)3
31P CDCl3
29.86
7.28
0.68
0.14
170 160 150 140 130 120 110 100 90 80 70 60 50 40 30 20 10 0 -10 -20 -30 -40 -50 -60 -70
ppm

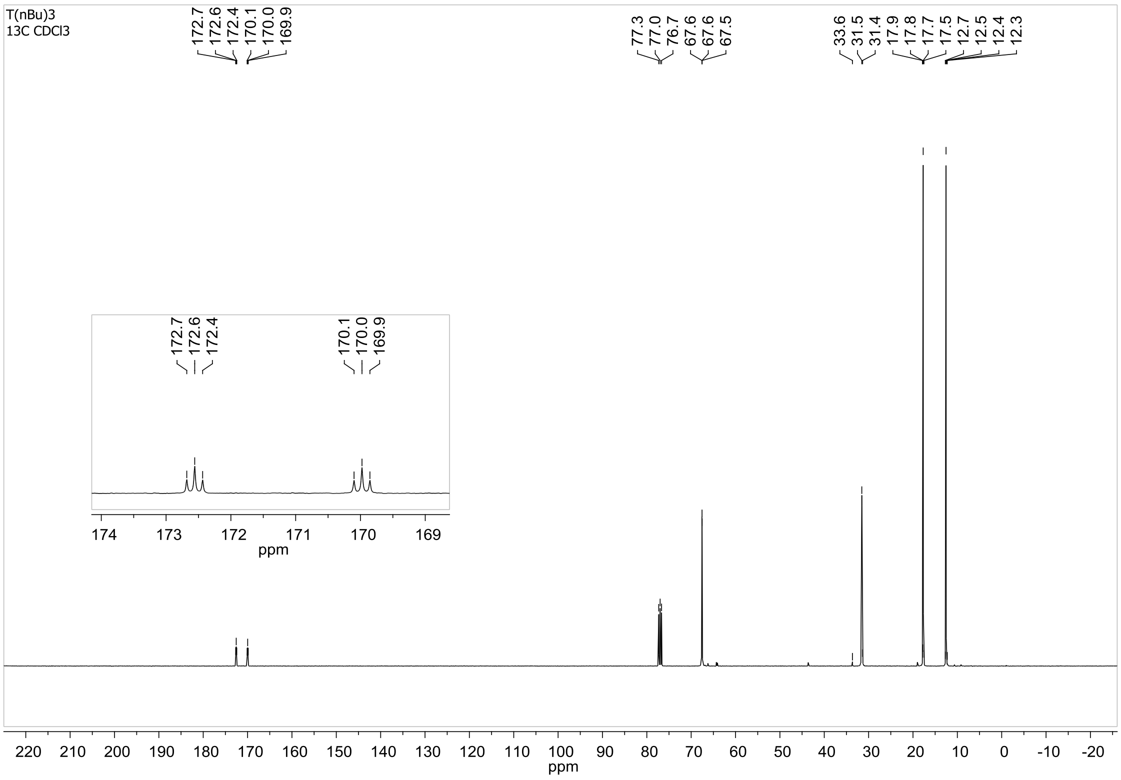

T(nBu)3
13C CDCl3
172.7
172.6
172.4
170.1
170.0
169.9
77.3
77.0
76.7
67.6
67.6
67.5
33.6
31.5
31.4
17.9
17.8
17.7
17.5
12.7
12.5
12.4
12.3
174 173 172 171 170 169
ppm
220 210 200 190 180 170 160 150 140 130 120 110 100 90 80 70 60 50 40 30 20 10 0 -10 -20
ppm

Figure S5: NMR spectra of 2,4,6-tris(di-*iso*-decylphosphonate)-1,3,5-triazine T(iDec)$_3$: a) ^{1}H, b) ^{31}P and c) ^{13}C

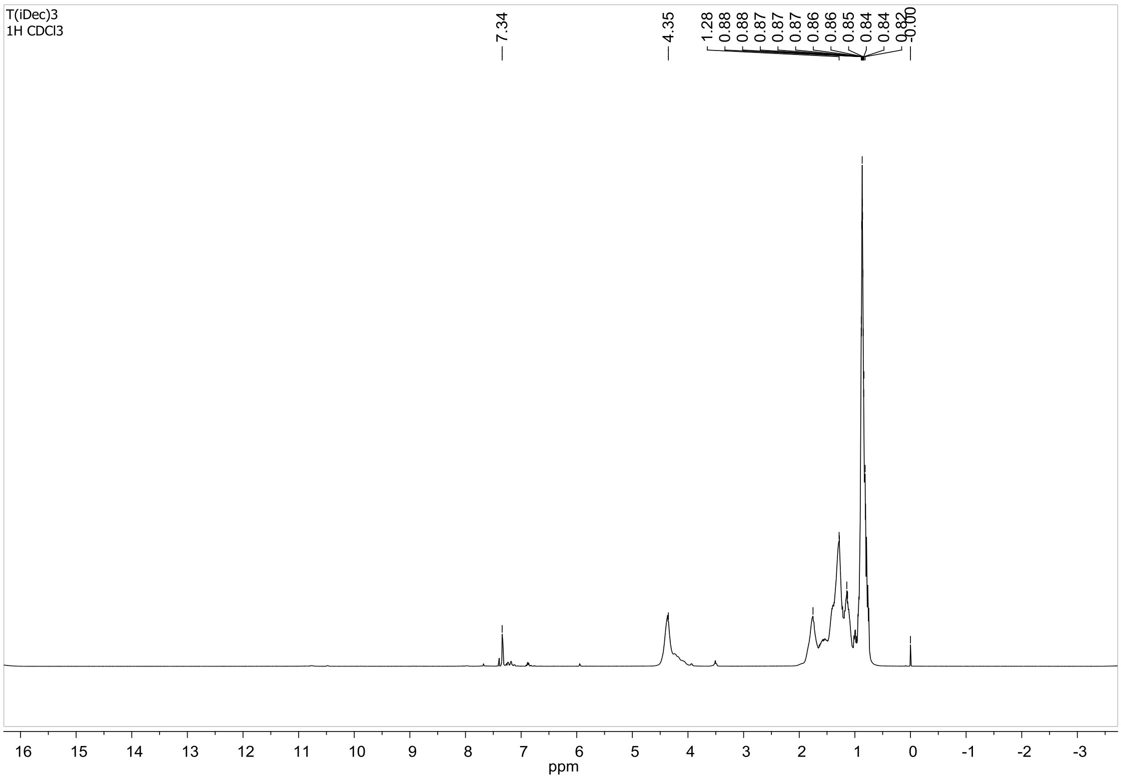

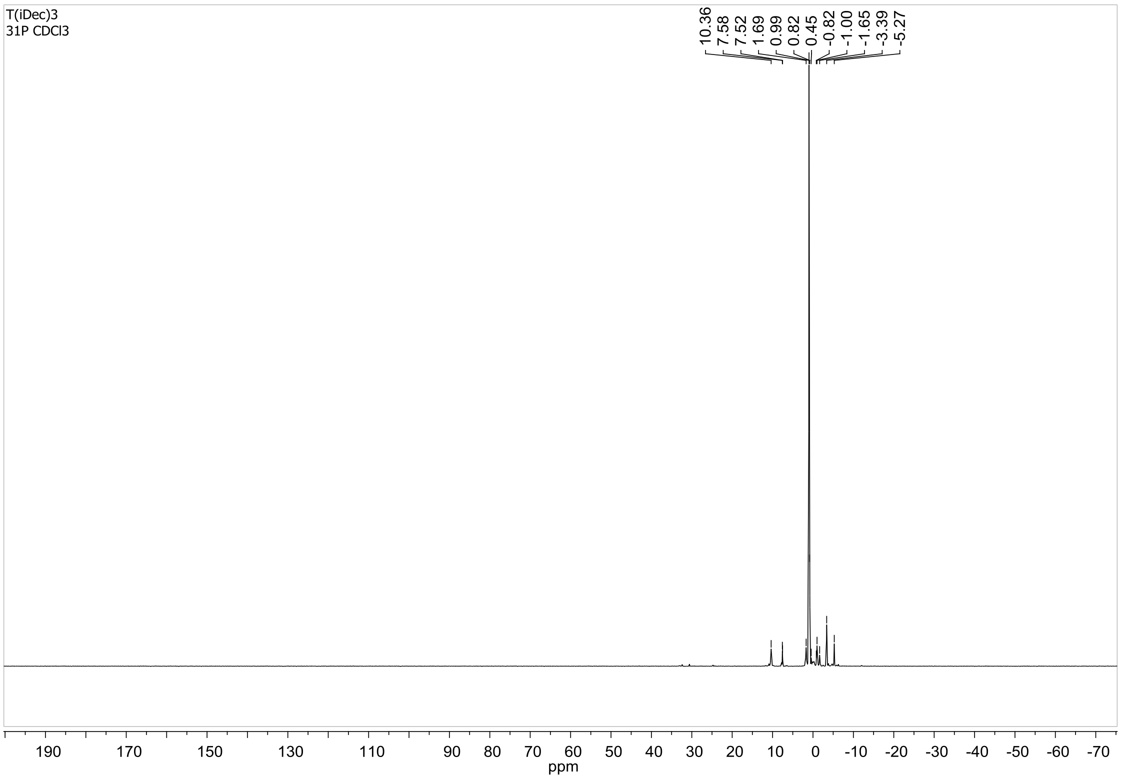
T(iDec)3
31P CDCl3
10.36
7.58
7.52
1.69
0.99
0.82
0.45
-0.82
-1.00
-1.65
-3.39
-5.27
ppm

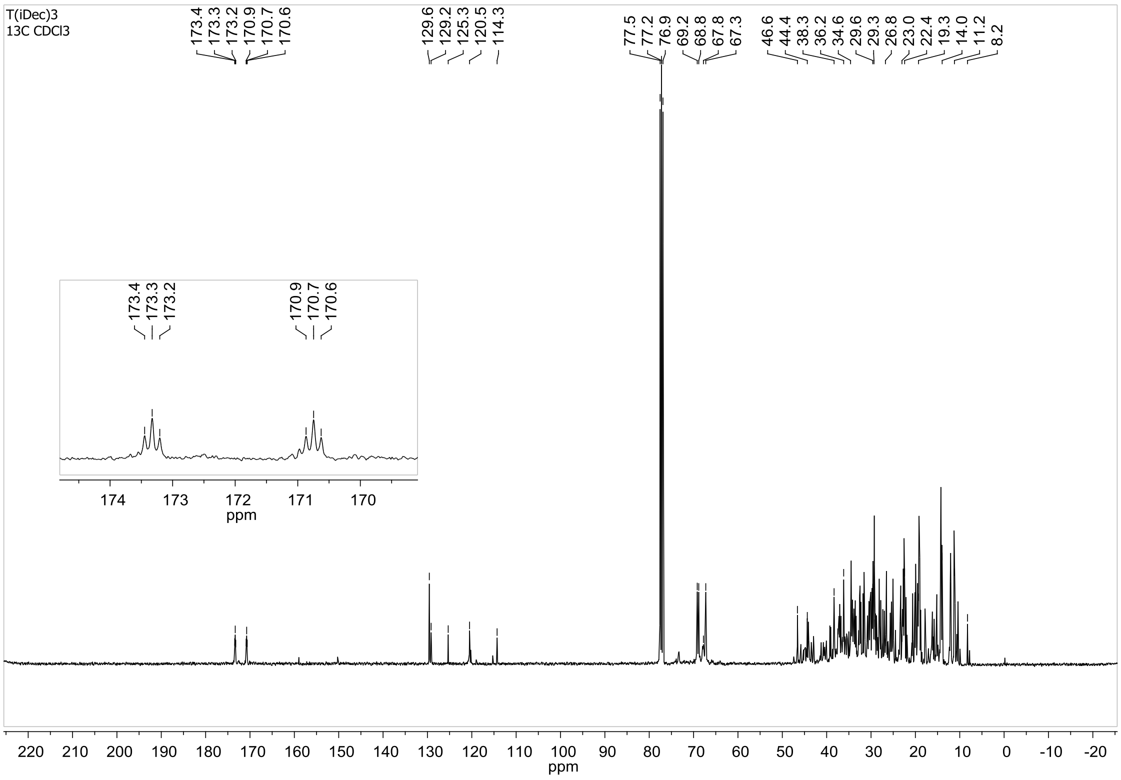
T(iDec)3
13C CDCl3
173.4
173.3
173.2
170.9
170.7
170.6
129.6
129.2
125.3
120.5
114.3
77.5
77.2
76.9
69.2
68.8
67.8
67.3
46.6
44.4
38.3
36.2
34.6
29.6
29.3
26.8
23.0
22.4
19.3
14.0
11.2
8.2
ppm

Figure S6: NMR spectra of the mixture mT/Me/2nBu: a) ^{1}H, b) ^{31}P and c) ^{13}C

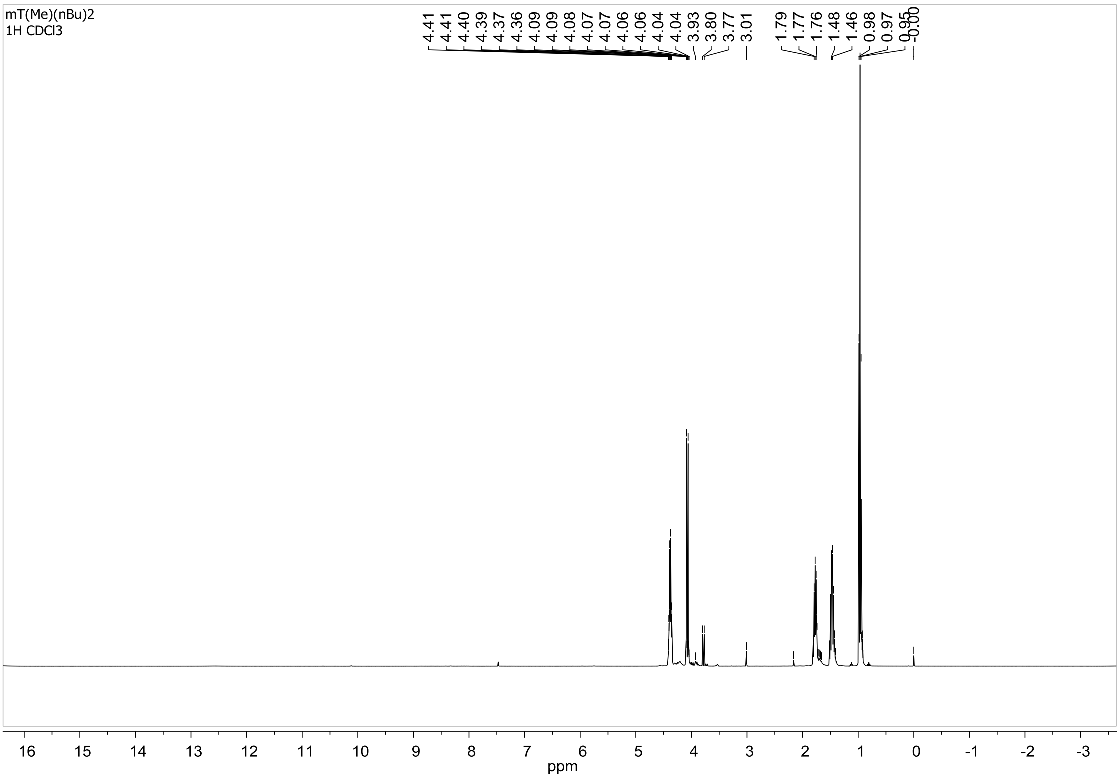

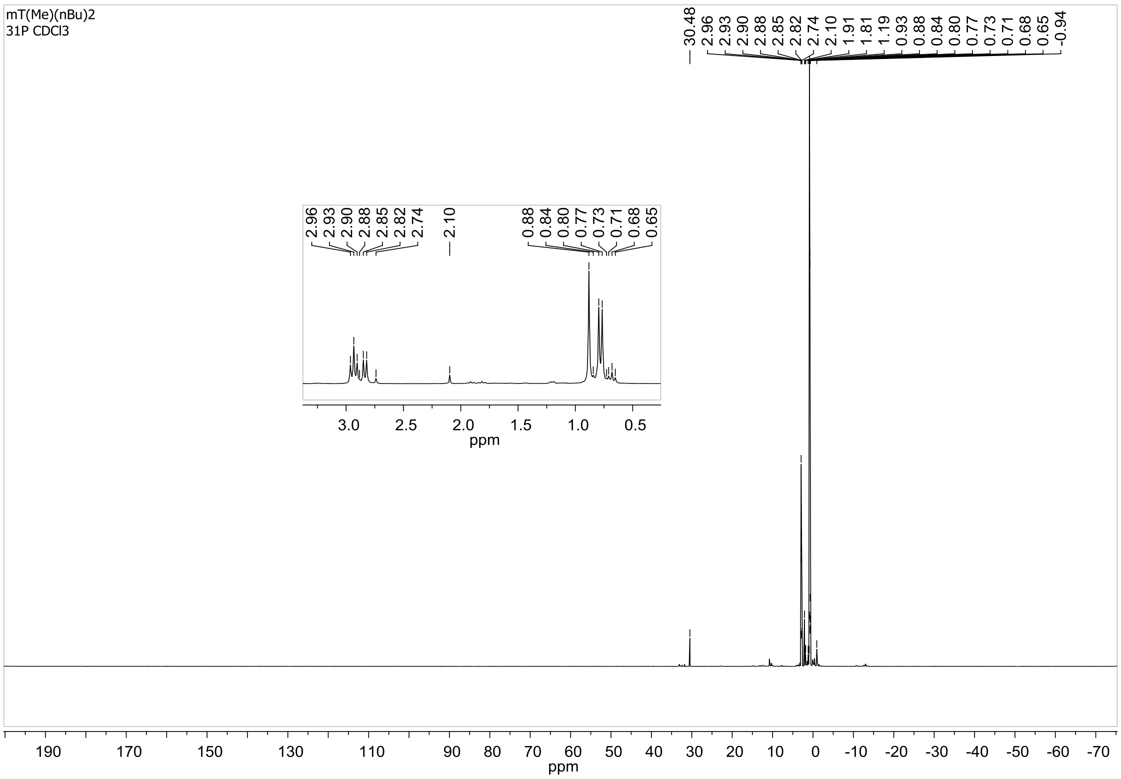
mT(Me)(nBu)2
31P CDCl3
30.48
2.96
2.93
2.90
2.88
2.85
2.82
2.74
2.10
1.91
1.81
1.19
0.93
0.88
0.84
0.80
0.77
0.73
0.71
0.68
0.65
-0.94
ppm

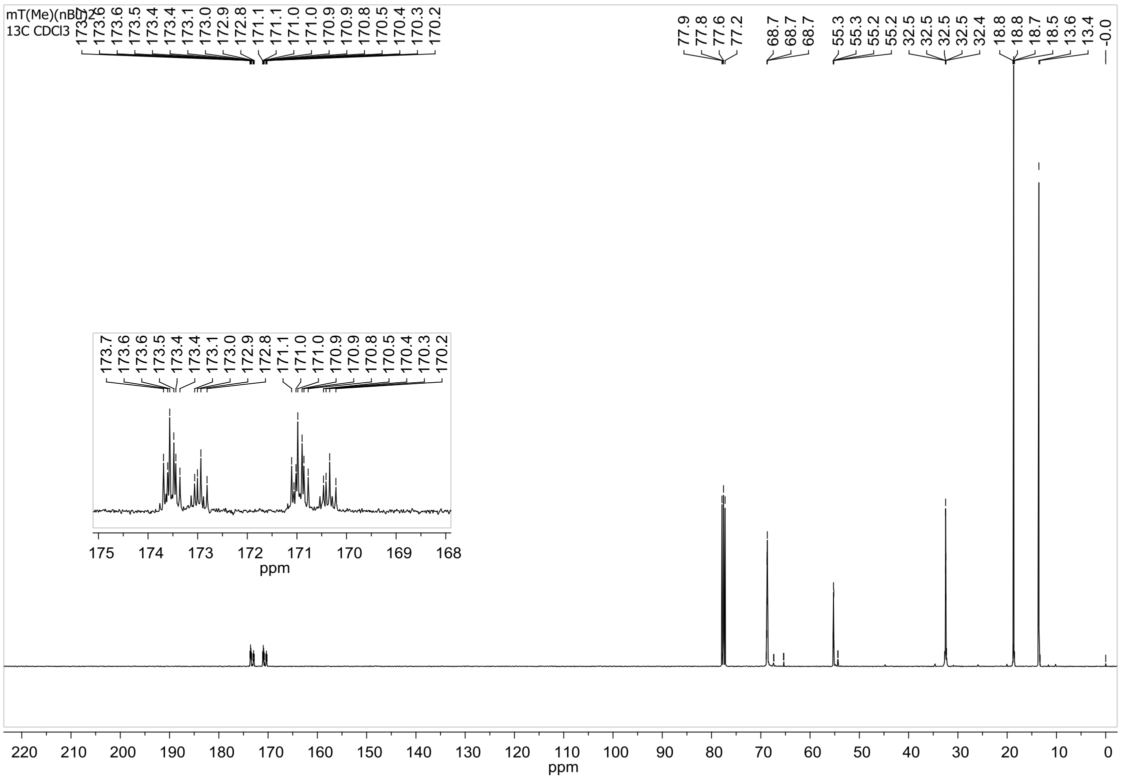
mT(Me)(nBu)2
13C CDCl3
77.9
77.8
77.6
77.2
68.7
68.7
68.7
55.3
55.3
55.2
55.2
32.5
32.5
32.5
32.5
32.4
18.8
18.8
18.7
18.5
13.6
13.4
-0.0
ppm

Figure S7: NMR spectra of the mixture mT/iPr/2nBu: a) ^{1}H, b) ^{31}P and c) ^{13}C

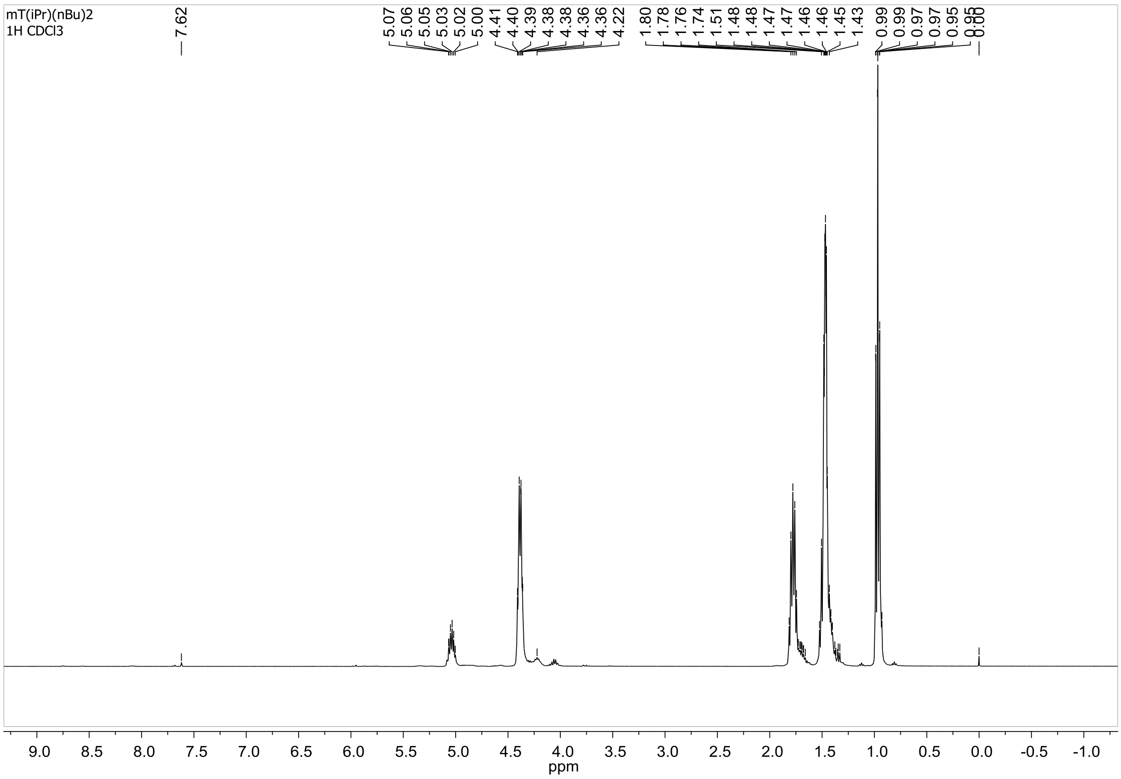

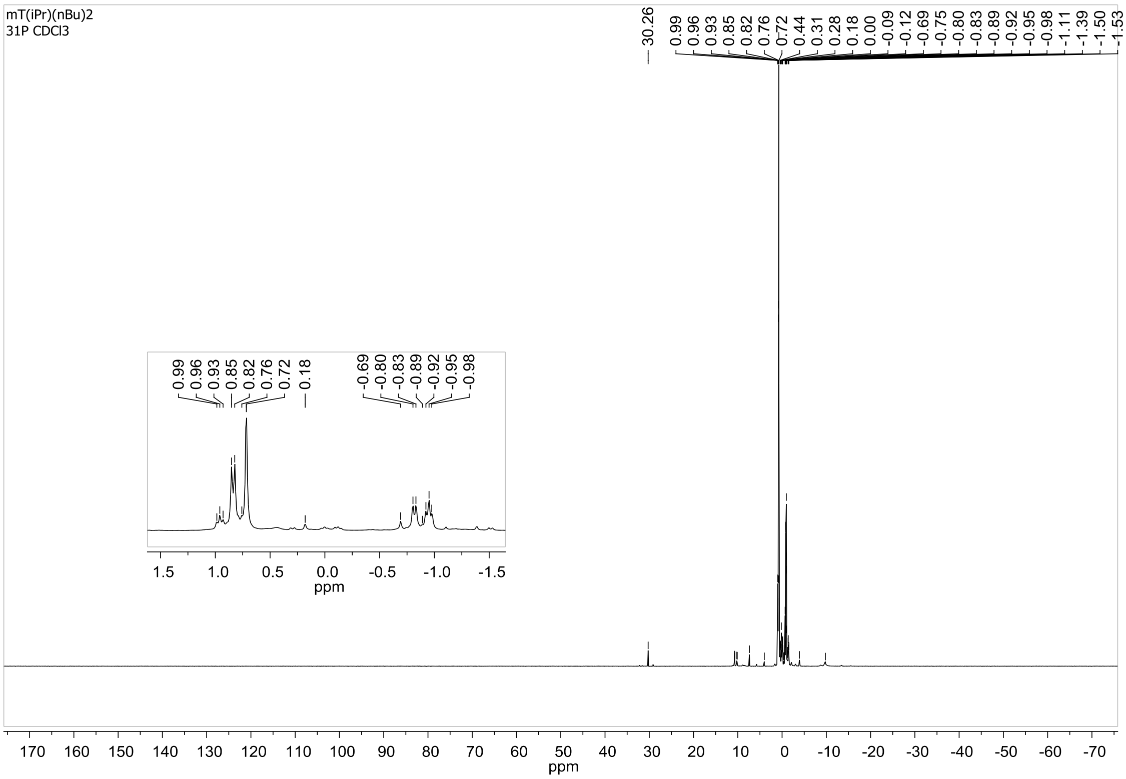
mT(iPr)(nBu)2
31P CDCl3
30.26
0.99 0.96 0.93 0.85 0.82 0.76 0.72 0.44 0.31 0.28 0.18 0.00 -0.09 -0.12 -0.69 -0.75 -0.80 -0.83 -0.89 -0.92 -0.95 -0.98 -1.11 -1.39 -1.50 -1.53
ppm

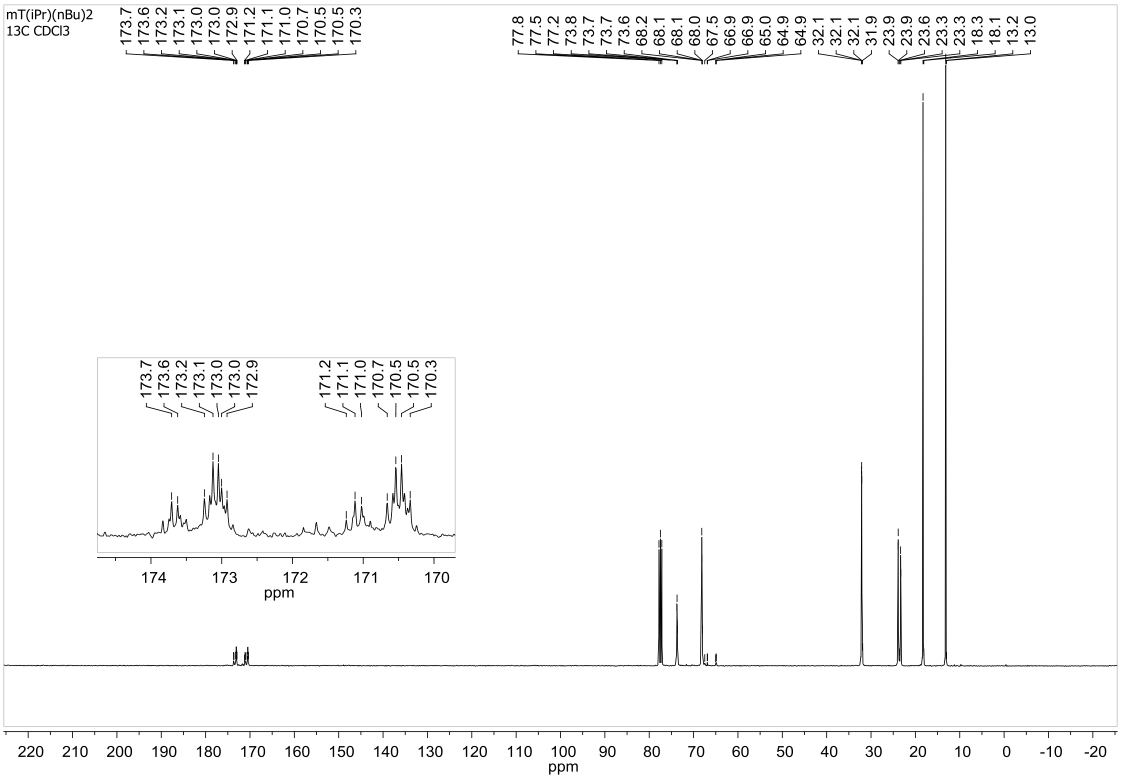
mT(iPr)(nBu)2
13C CDCl3
173.7 173.6 173.2 173.1 173.0 173.0 172.9 171.2 171.1 171.0 170.7 170.5 170.5 170.3
77.8 77.5 77.2 73.8 73.7 73.7 73.6 68.2 68.1 68.1 68.0 67.5 66.9 66.9 65.0 64.9 64.9 32.1 32.1 32.1 31.9 23.9 23.9 23.6 23.3 23.3 18.3 18.1 13.2 13.0
ppm

Figure S8: NMR spectra of the mixture mT/2iPr/nBu: a) ^{1}H, b) ^{31}P and c) ^{13}C

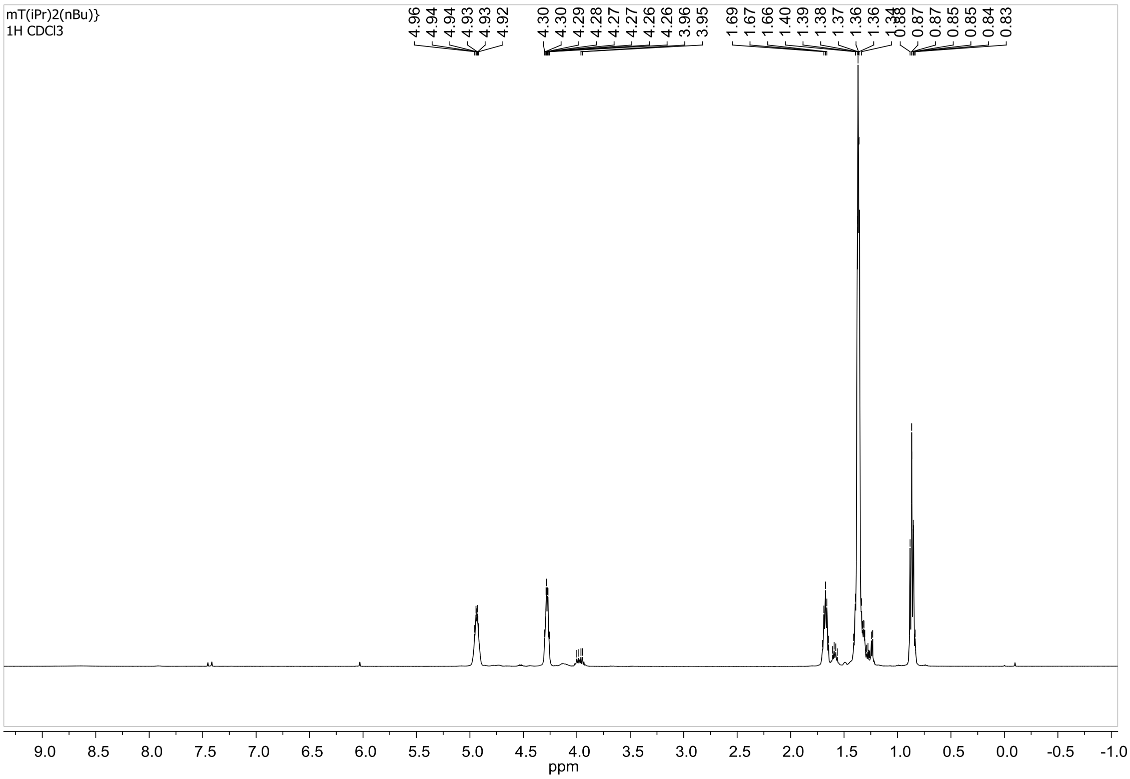

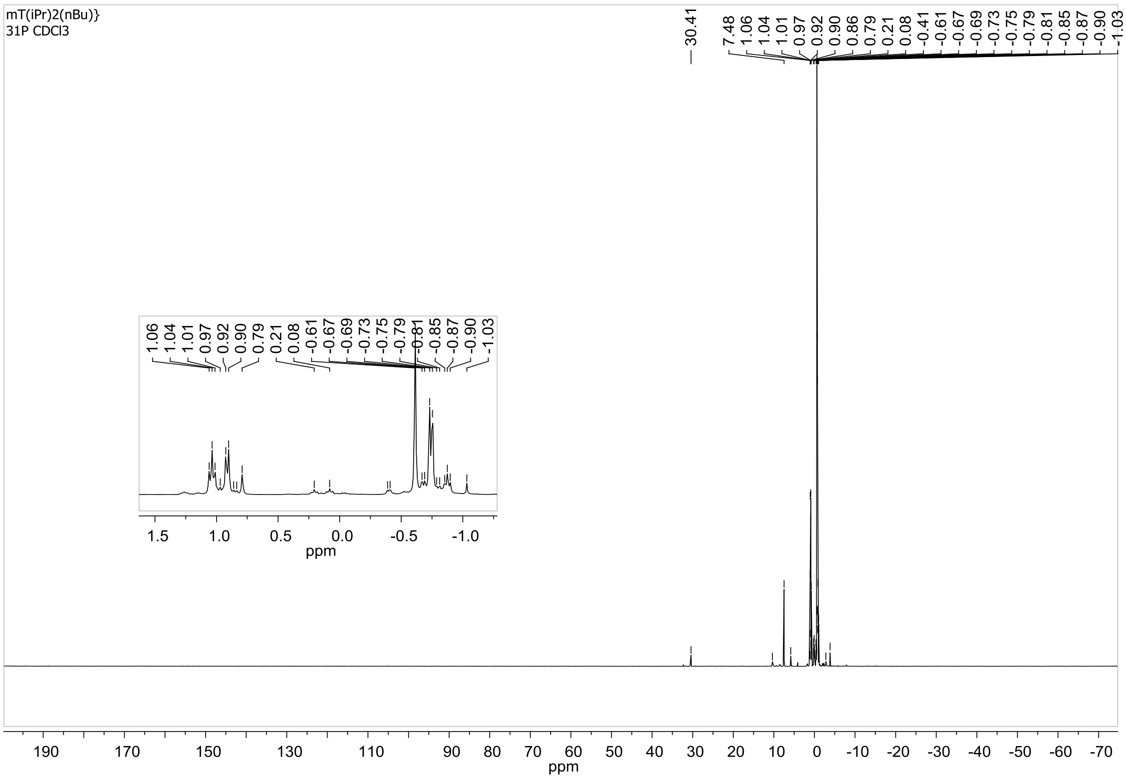
mT(iPr)2(nBu)}
31P CDCl3
30.41
7.48
1.06
1.04
1.01
0.97
0.92
0.90
0.86
0.79
0.21
0.08
-0.41
-0.61
-0.67
-0.69
-0.73
-0.75
-0.79
-0.81
-0.85
-0.87
-0.90
-1.03
ppm

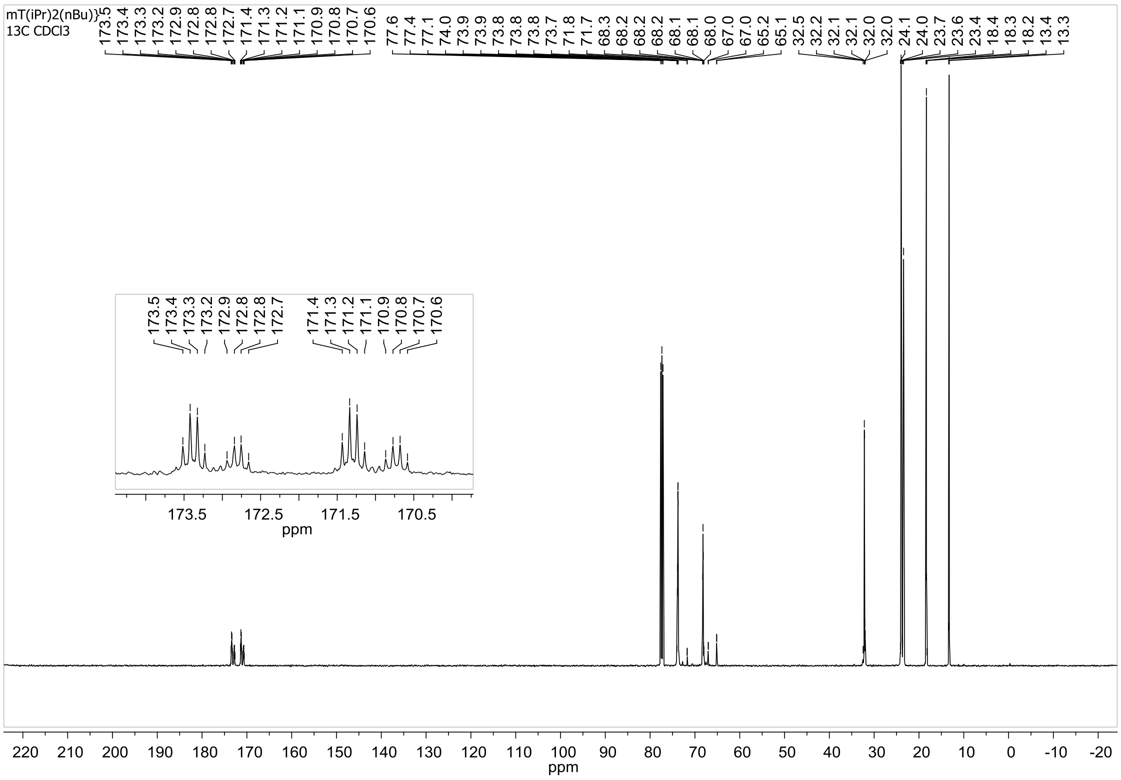
mT(iPr)2(nBu)}
13C CDCl3
173.5
173.4
173.3
173.2
172.9
172.8
172.8
172.7
171.4
171.3
171.2
171.1
170.9
170.8
170.7
170.6
77.6
77.4
77.1
74.0
73.9
73.8
73.8
73.8
73.7
71.8
71.7
68.3
68.2
68.2
68.2
68.1
68.1
68.0
67.0
67.0
65.2
65.1
32.5
32.2
32.1
32.1
32.0
32.0
24.1
24.0
23.7
23.6
23.4
18.4
18.3
18.2
13.4
13.3
ppm

Figure S9: NMR spectra of the mixture mT/Et/iPr/nBu: a) ^{1}H, b) ^{31}P and c) ^{13}C

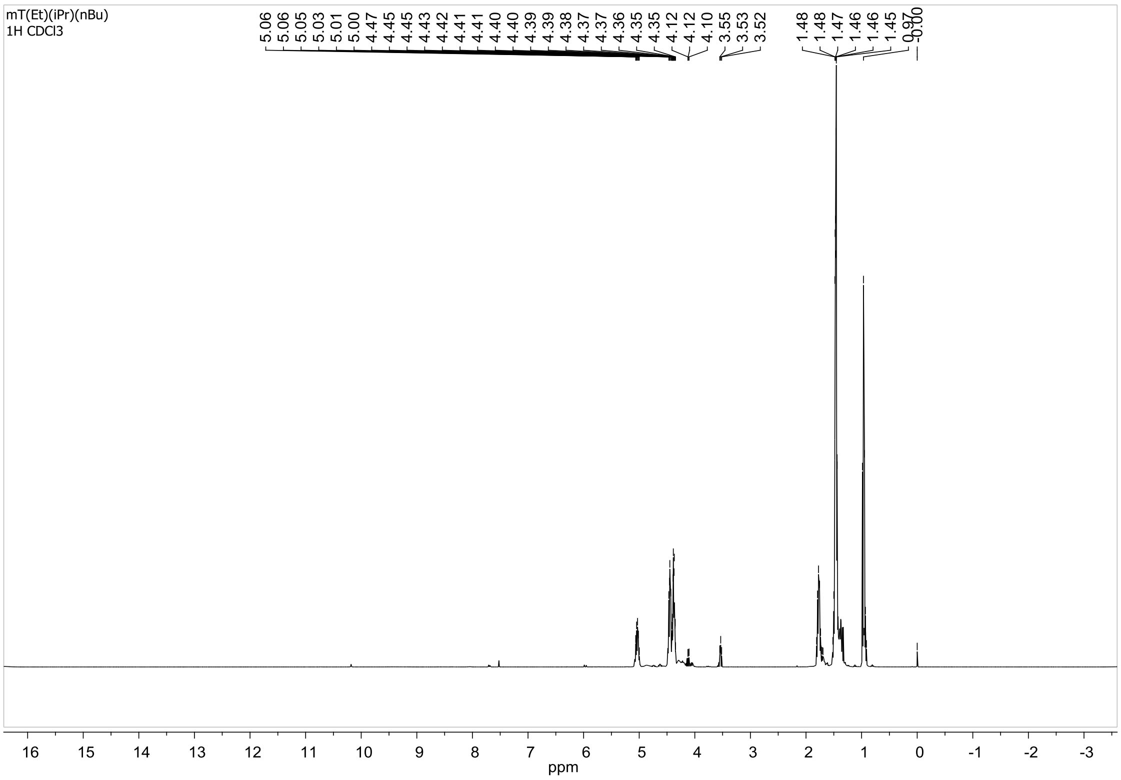

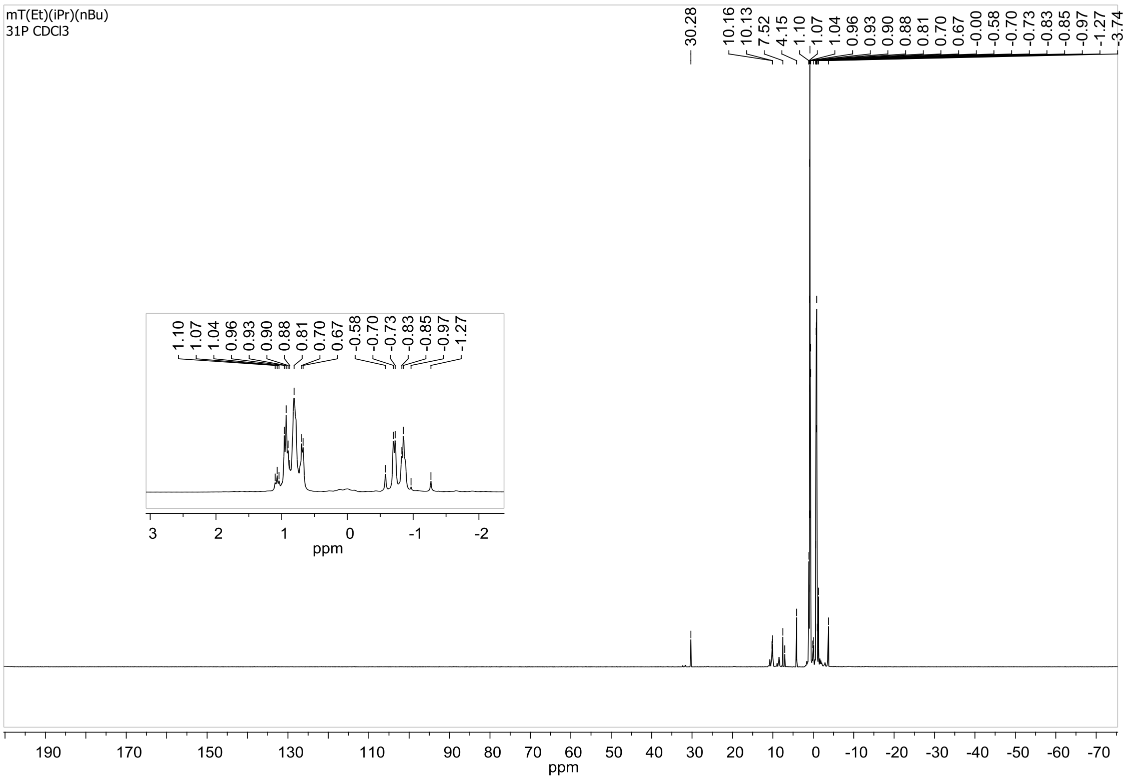

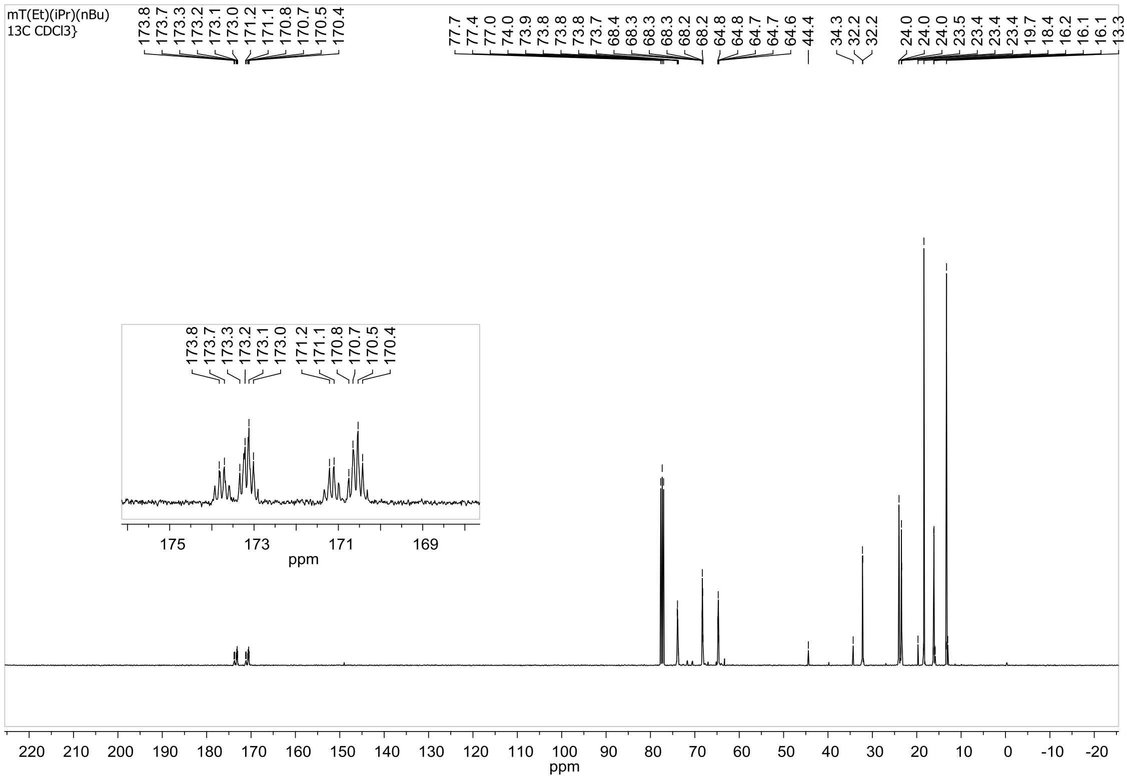
mT(Et)(iPr)(nBu)
13C CDCl3}
173.8 173.7 173.3 173.2 173.1 173.0 171.2 171.1 170.8 170.7 170.5 170.4
77.7 77.4 77.0 74.0 73.9 73.8 73.8 73.8 73.7 68.4 68.3 68.3 68.3 68.2 68.2 64.8 64.8 64.7 64.7 64.6 44.4 34.3 32.2 32.2 24.0 24.0 24.0 23.5 23.4 23.4 23.4 19.7 18.4 16.2 16.1 13.3
175 173 171 169
ppm
220 210 200 190 180 170 160 150 140 130 120 110 100 90 80 70 60 50 40 30 20 10 0 -10 -20
ppm

Figure S10: NMR spectra of the mixture mT/Me/Et/iPr: a) ^{1}H, b) ^{31}P and c) ^{13}C

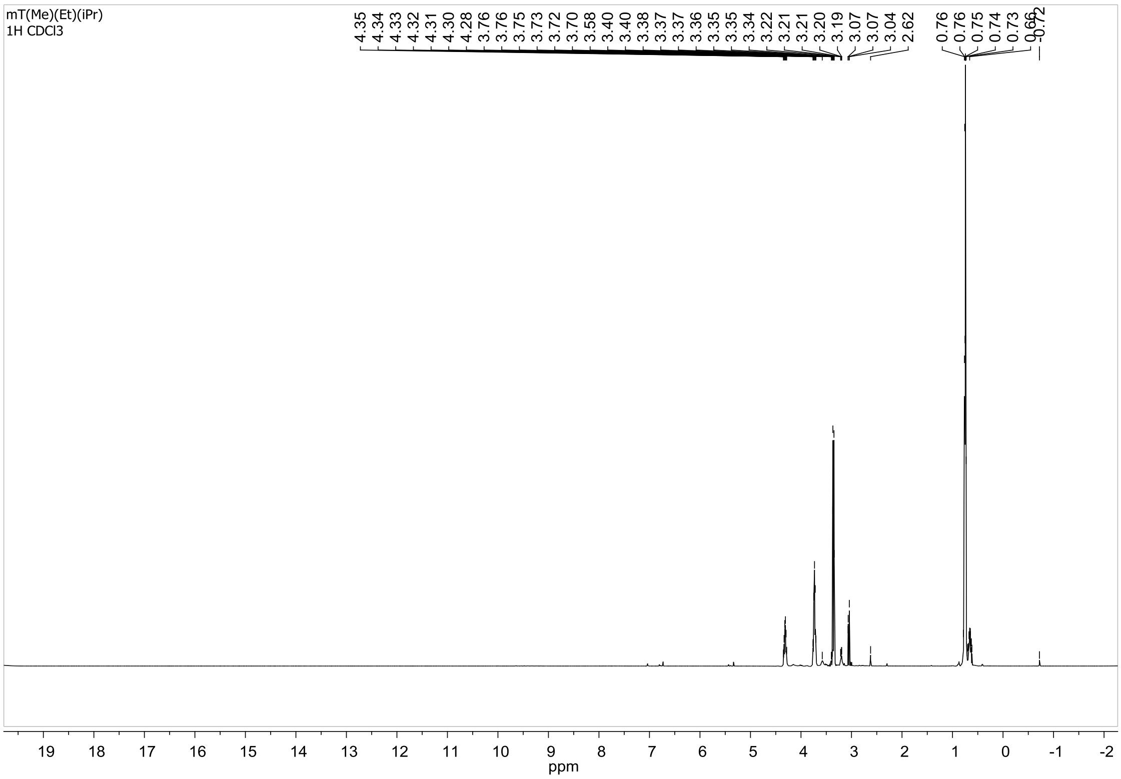

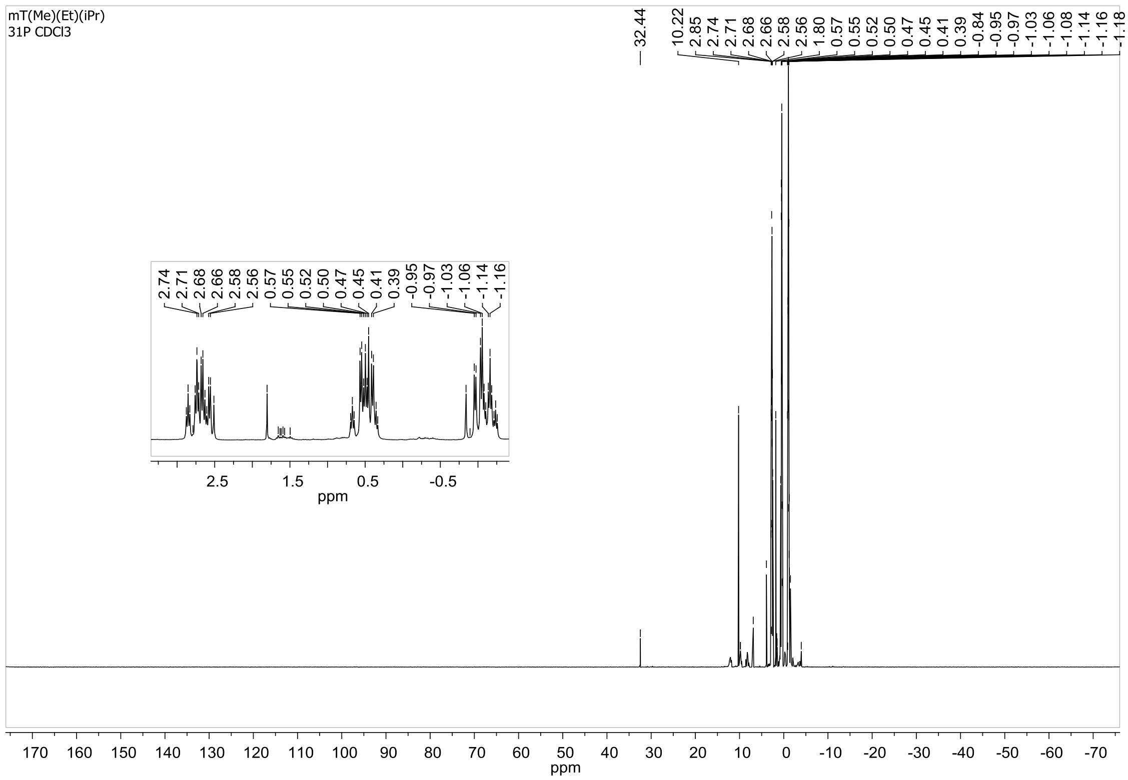
mT(Me)(Et)(iPr)
31P CDCl3
ppm

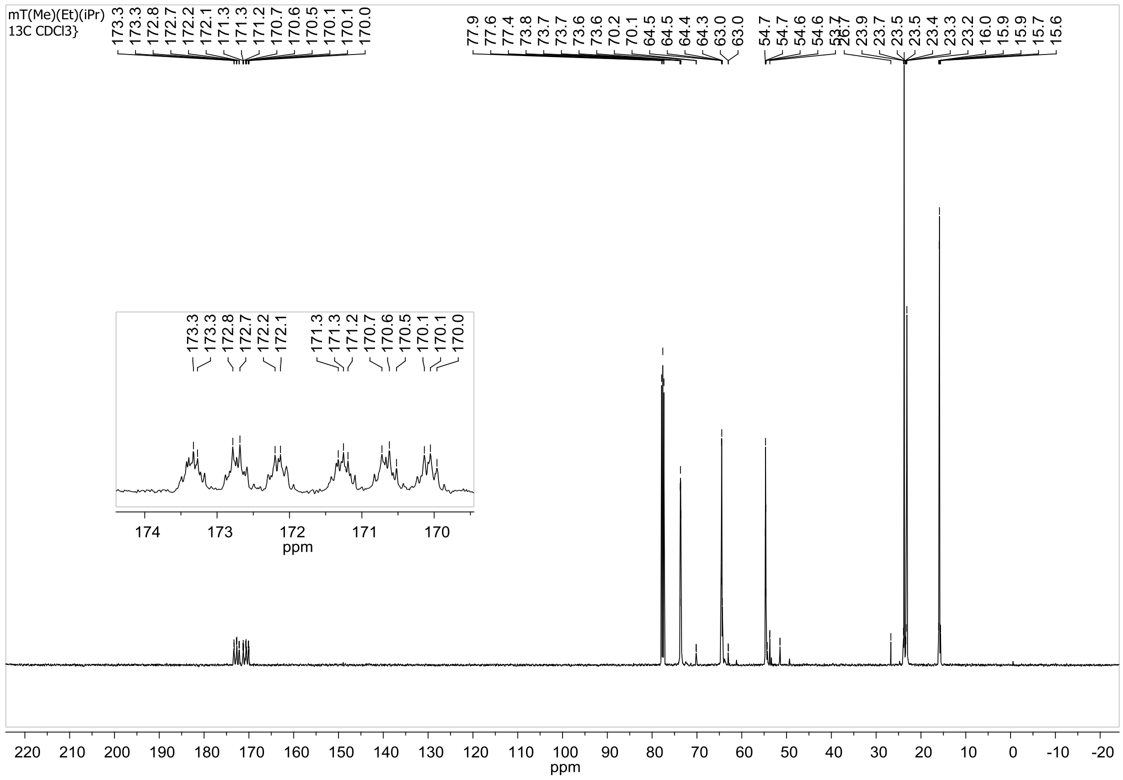
mT(Me)(Et)(iPr)
13C CDCl3}
ppm

Figure S11: NMR spectra of the mixture mT/Me/iPr/nBu: a) ^{1}H, b) ^{31}P and c) ^{13}C

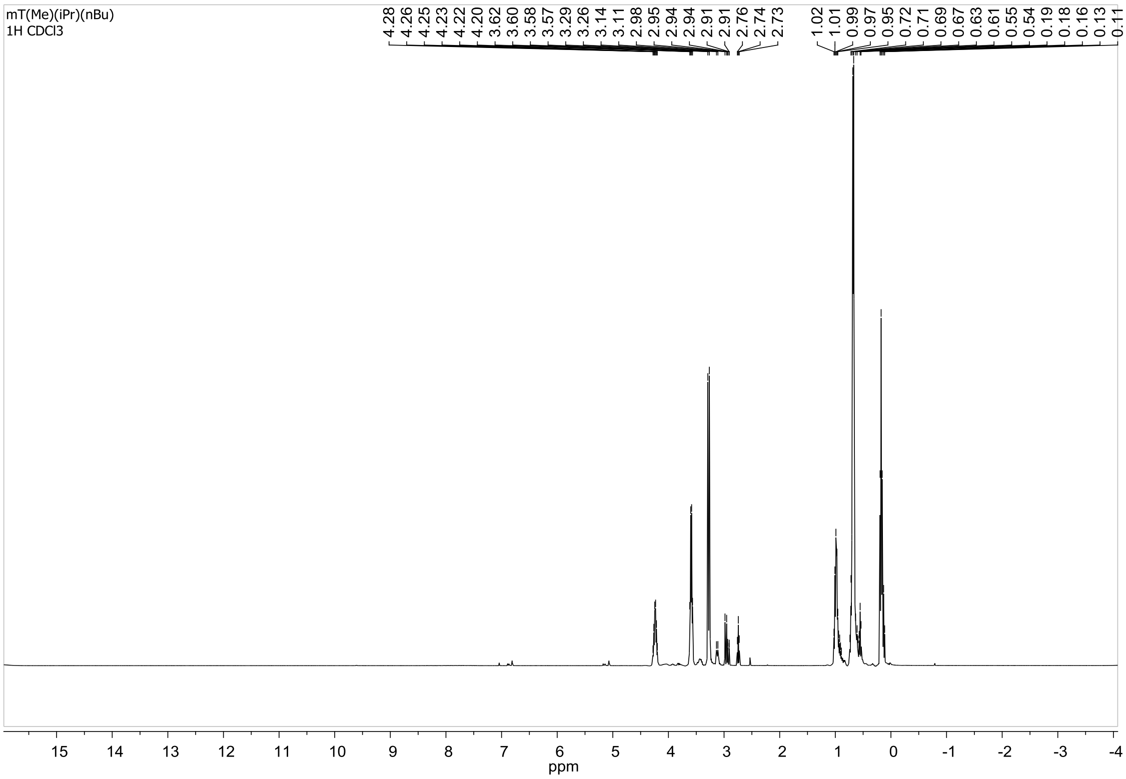

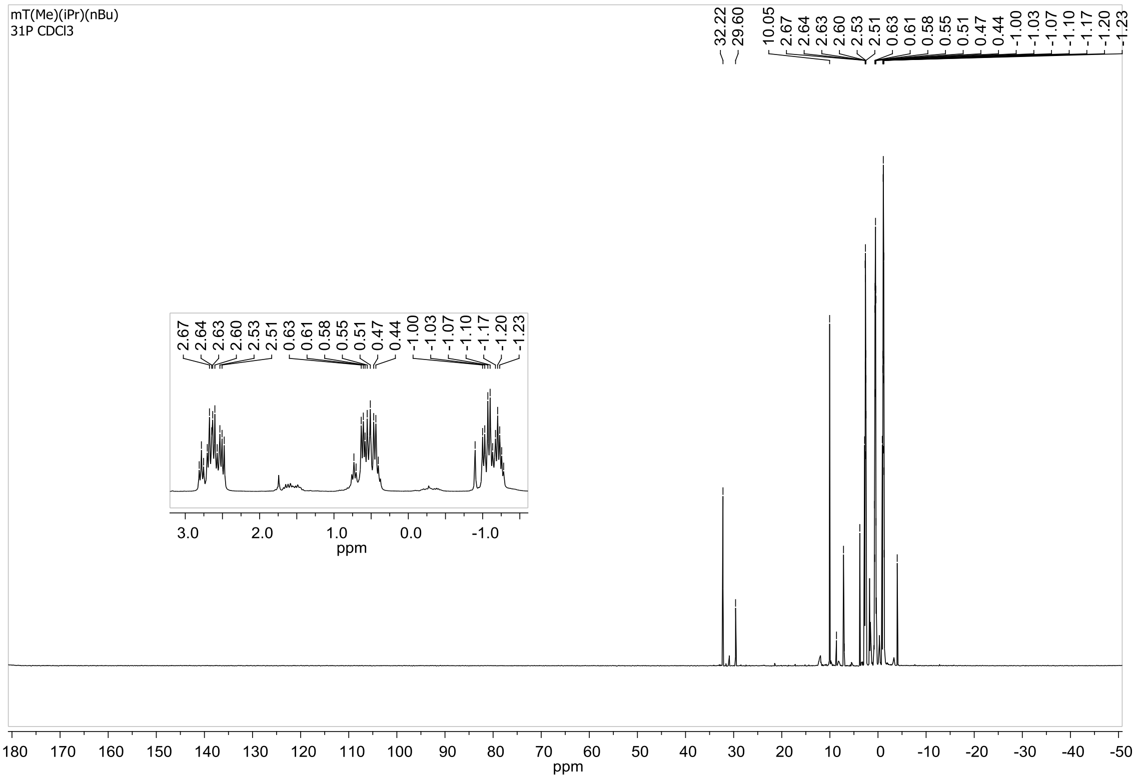
mT(Me)(iPr)(nBu)
31P CDCl3
ppm

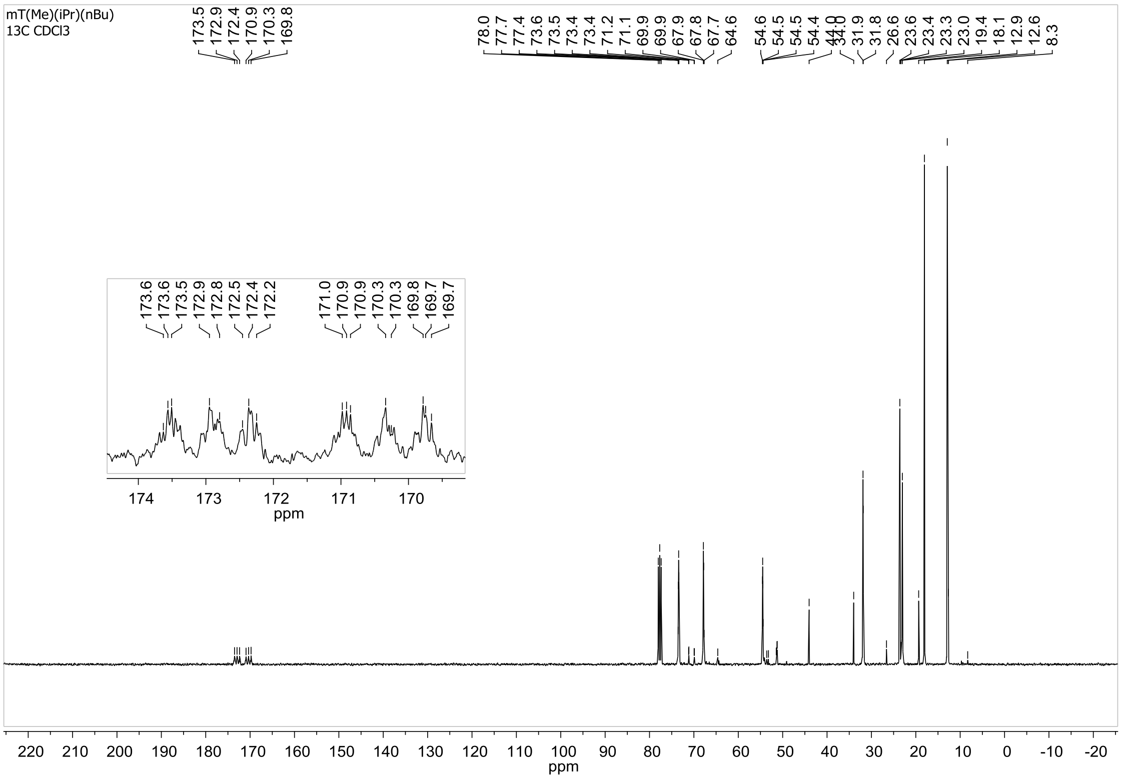
mT(Me)(iPr)(nBu)
13C CDCl3
ppm

Figure S12: Viscosity measurements at 20 °C of 2,4,6-tris(di-*n*-butylphosphonate)-1,3,5-triazine **T(nBu)$_3$ (B)** and the mixture **mT/Me/2nBu (C)**.

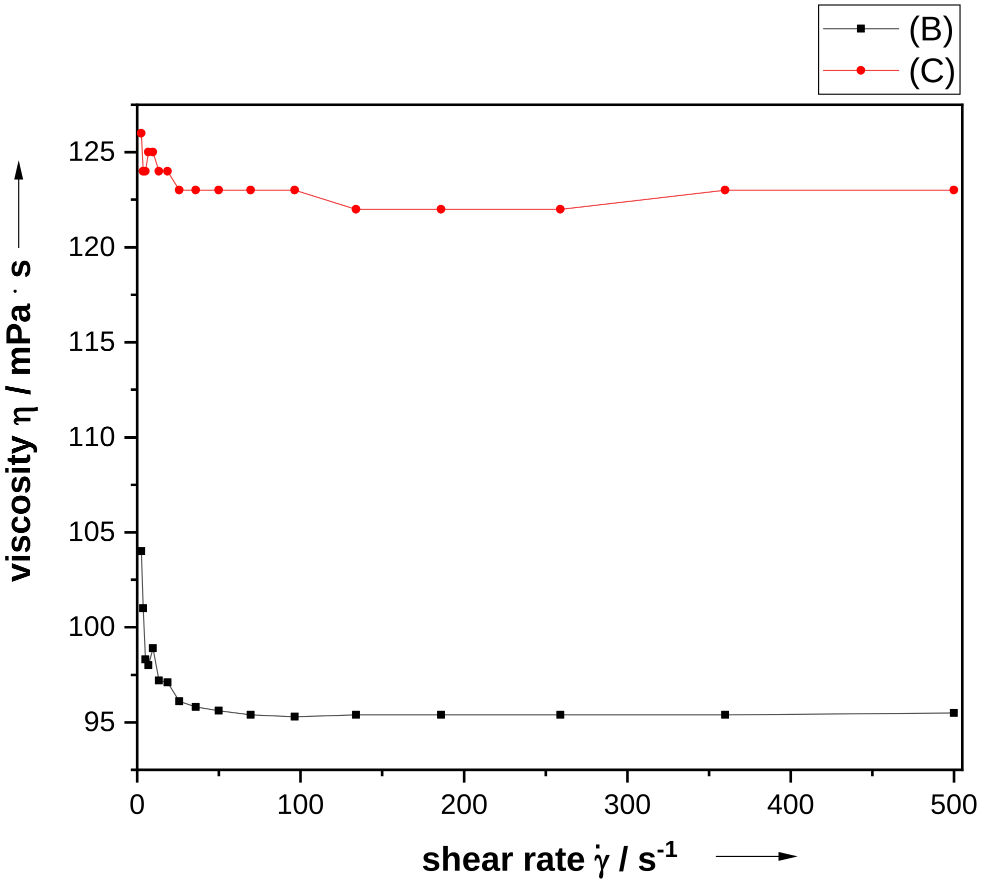

Fig. S13 ATR-IR spectrum of $T(Me)_3$

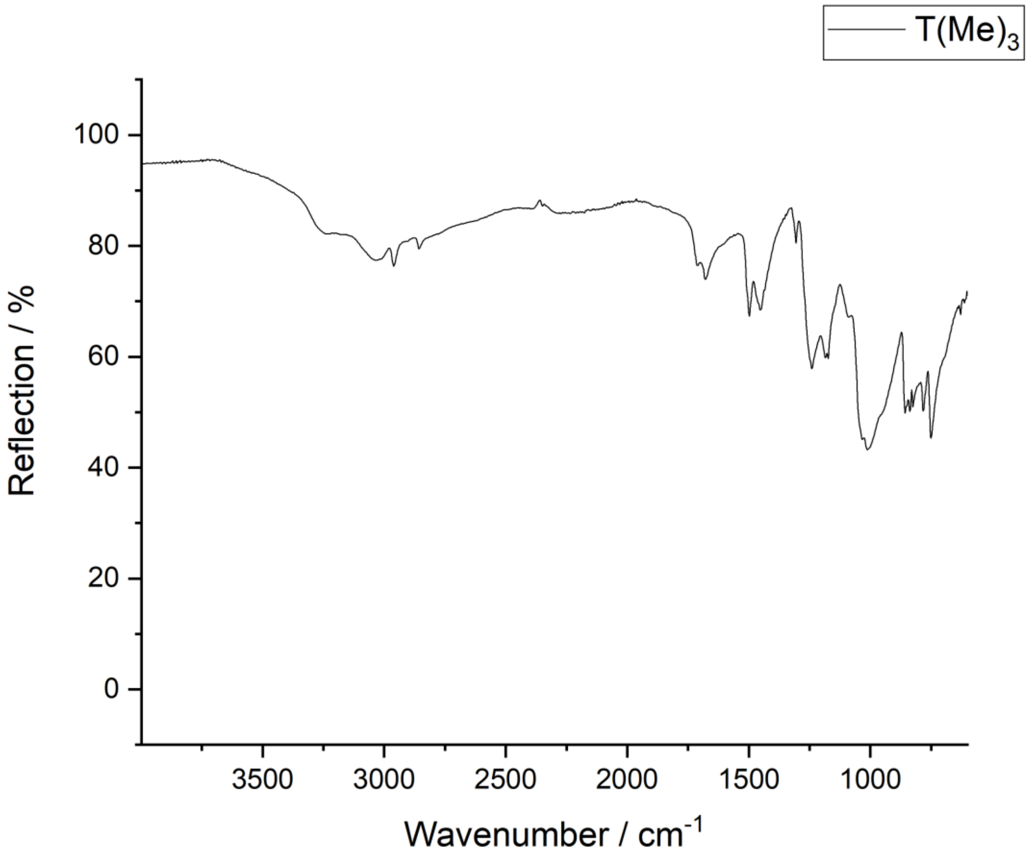

Fig. S14   ATR-IR spectrum of $T(Et)_3$

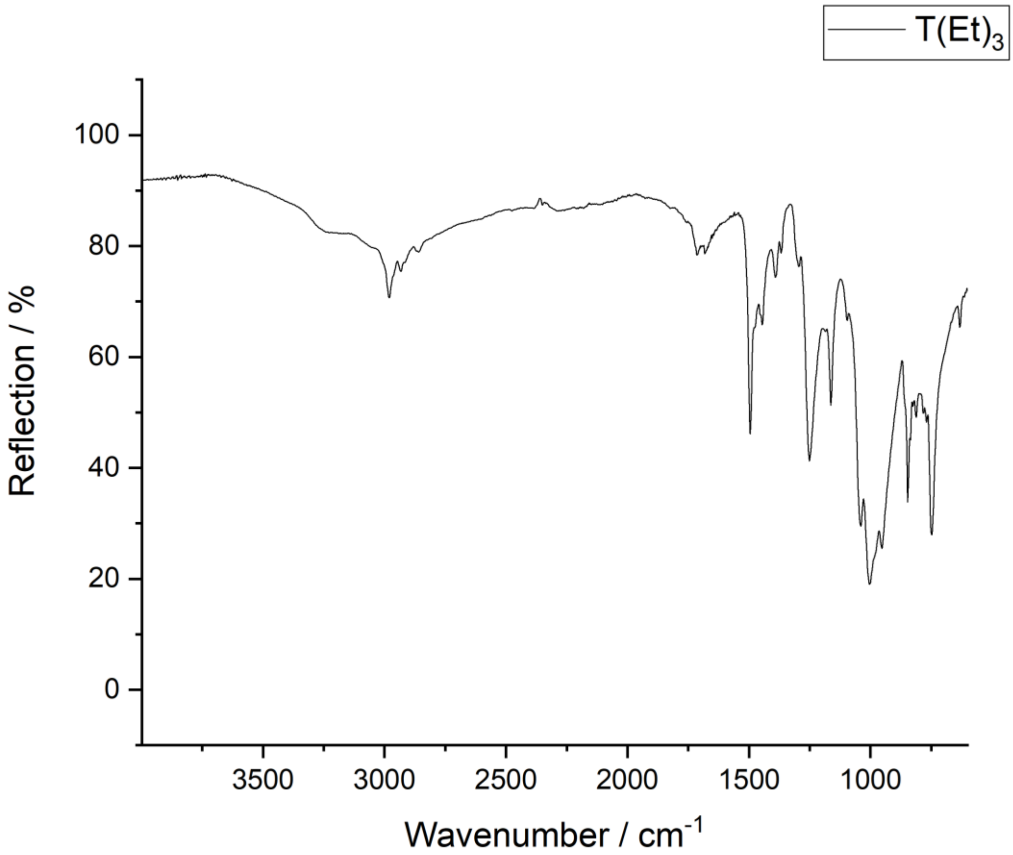

Fig. S15 ATR-IR spectrum of $T(iPr)_3$

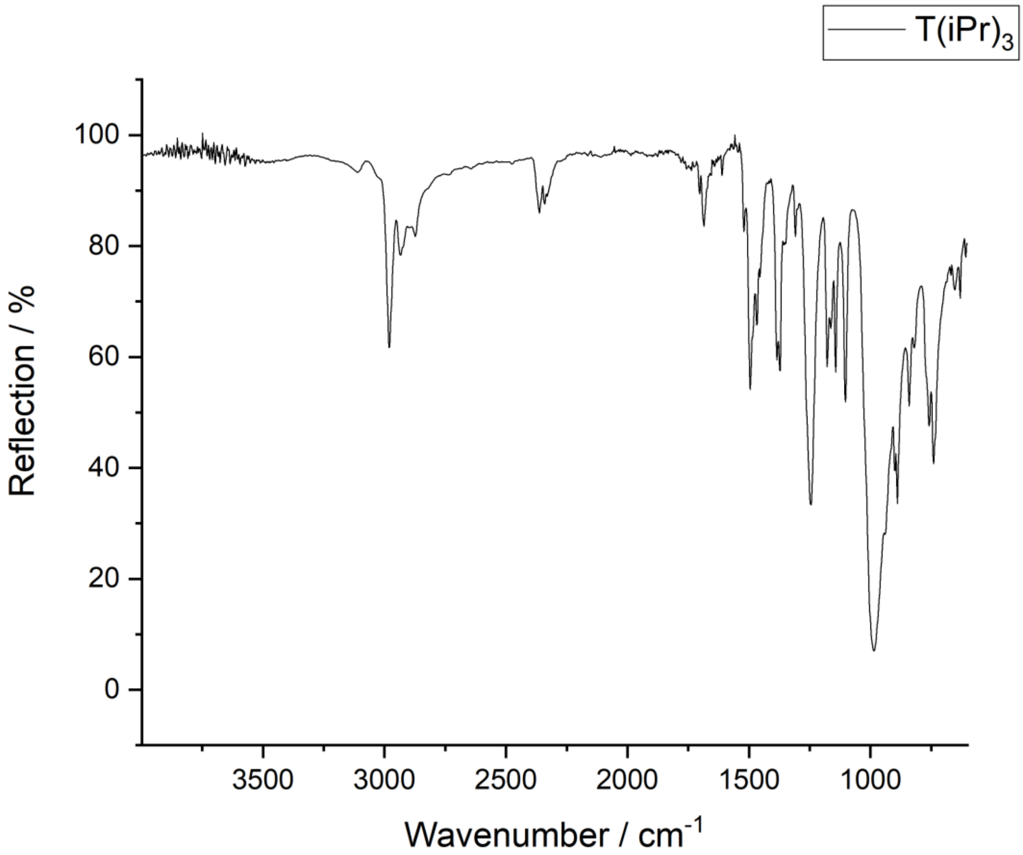

Fig. S16 ATR-IR spectrum of $T(nBu)_3$

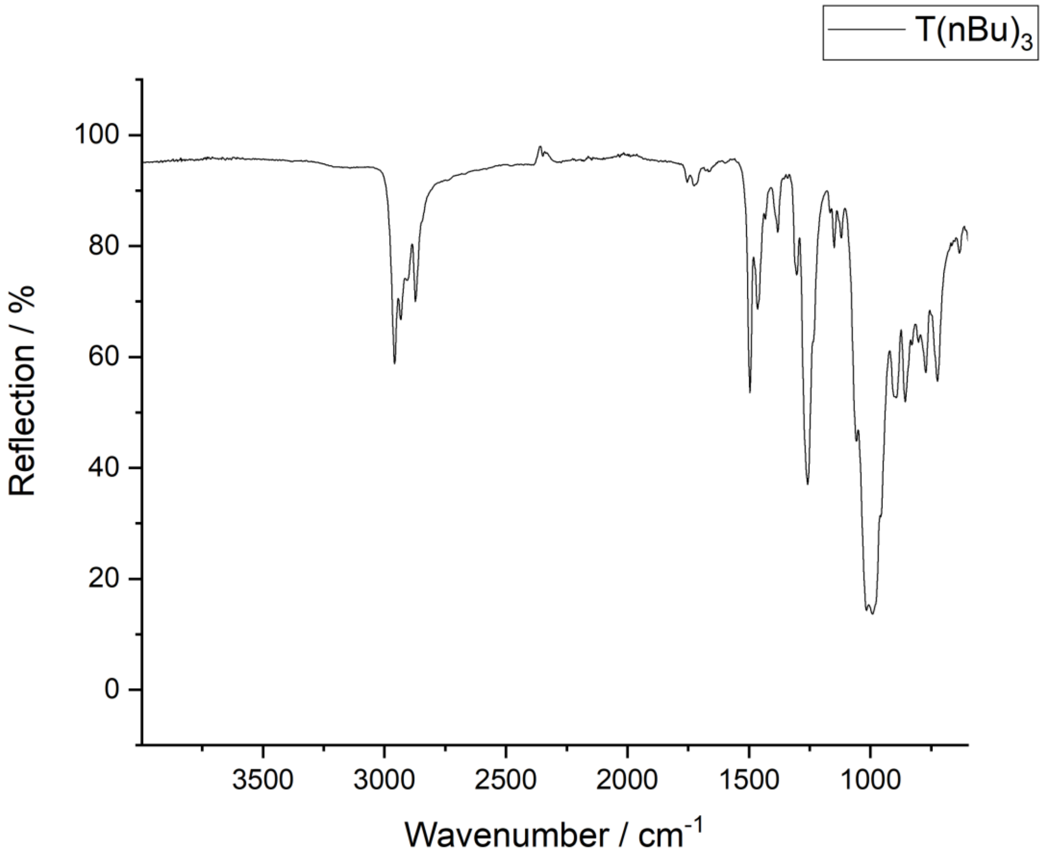

Fig. S17 ATR-IR spectrum of $T(iDec)_3$

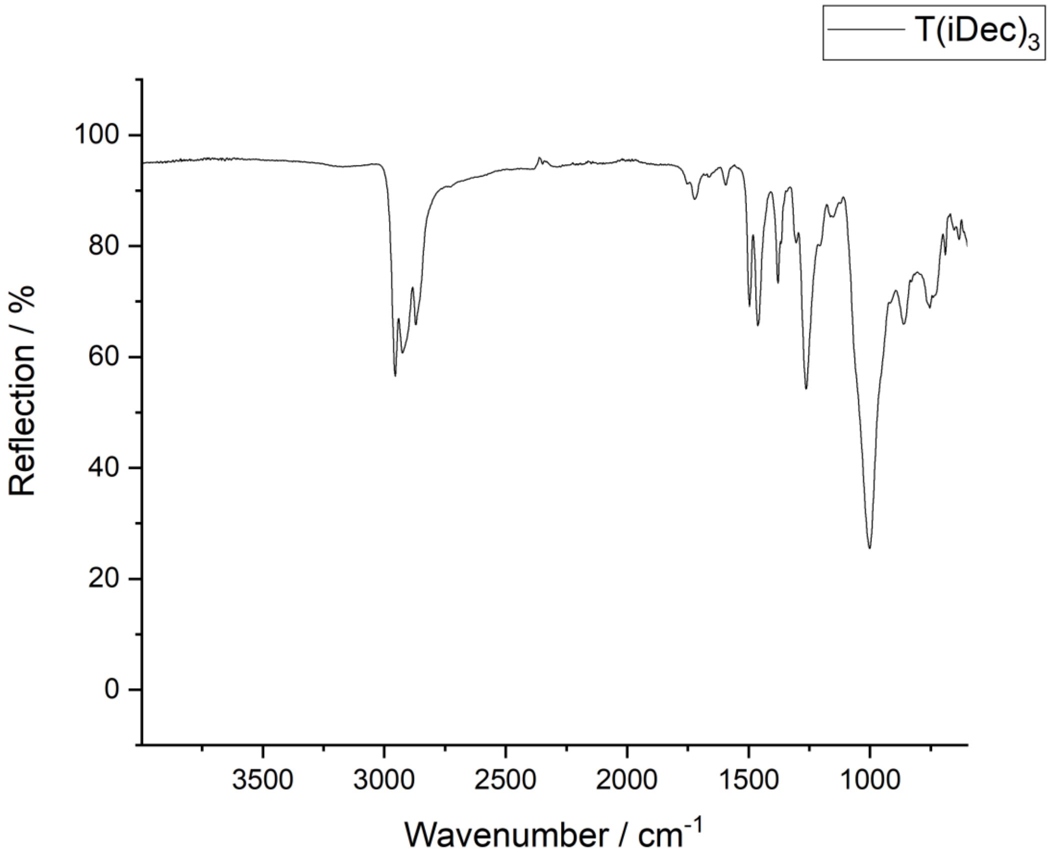

Fig. S18 ATR-IR spectrum of the mixture mT/Me/2nBu

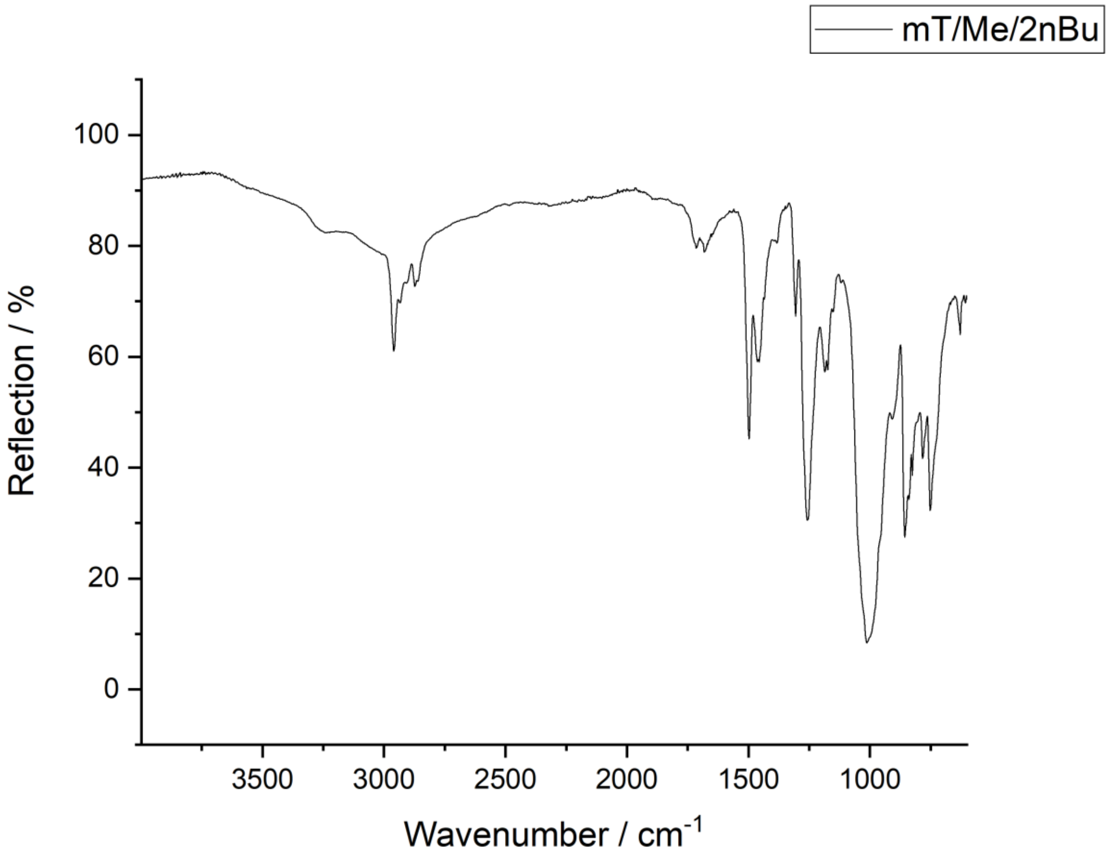

Fig. S7 **ATR-IR spectrum of the mixture mT/iPr/2nBu**

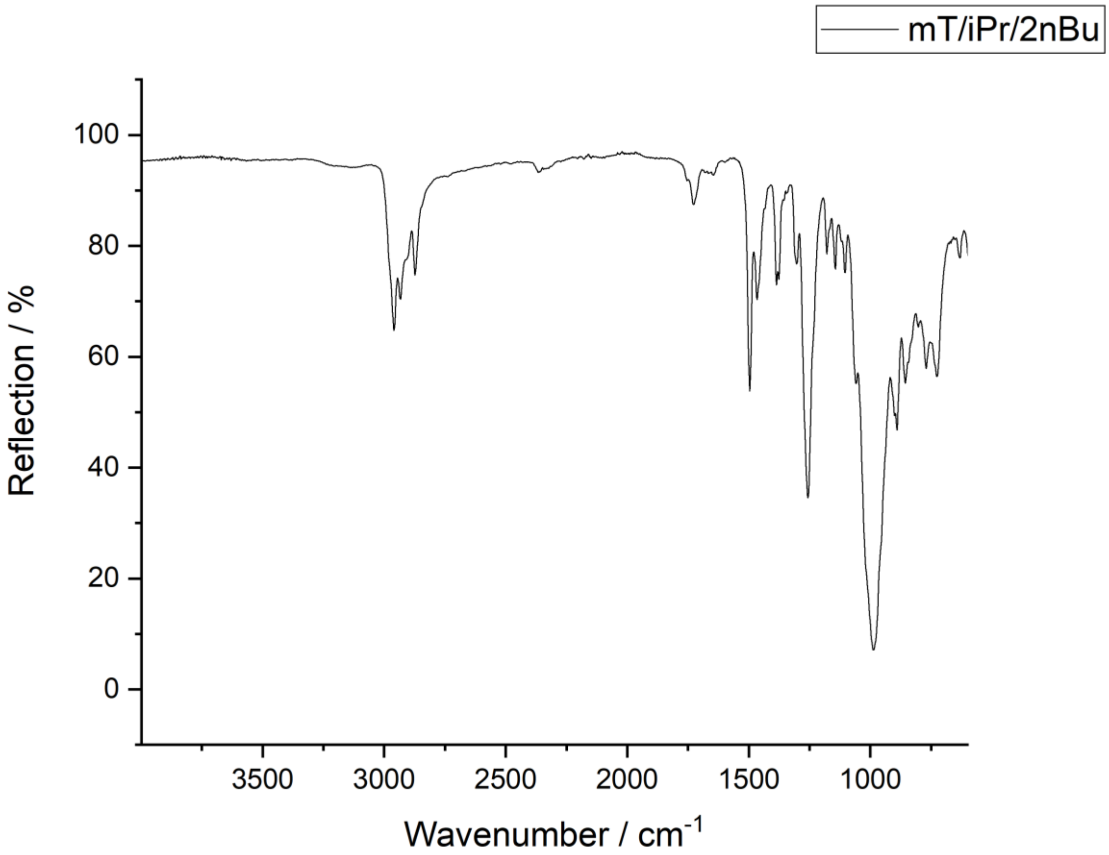

Fig. S20 ATR-IR spectrum of the mixture mT/2iPr/nBu

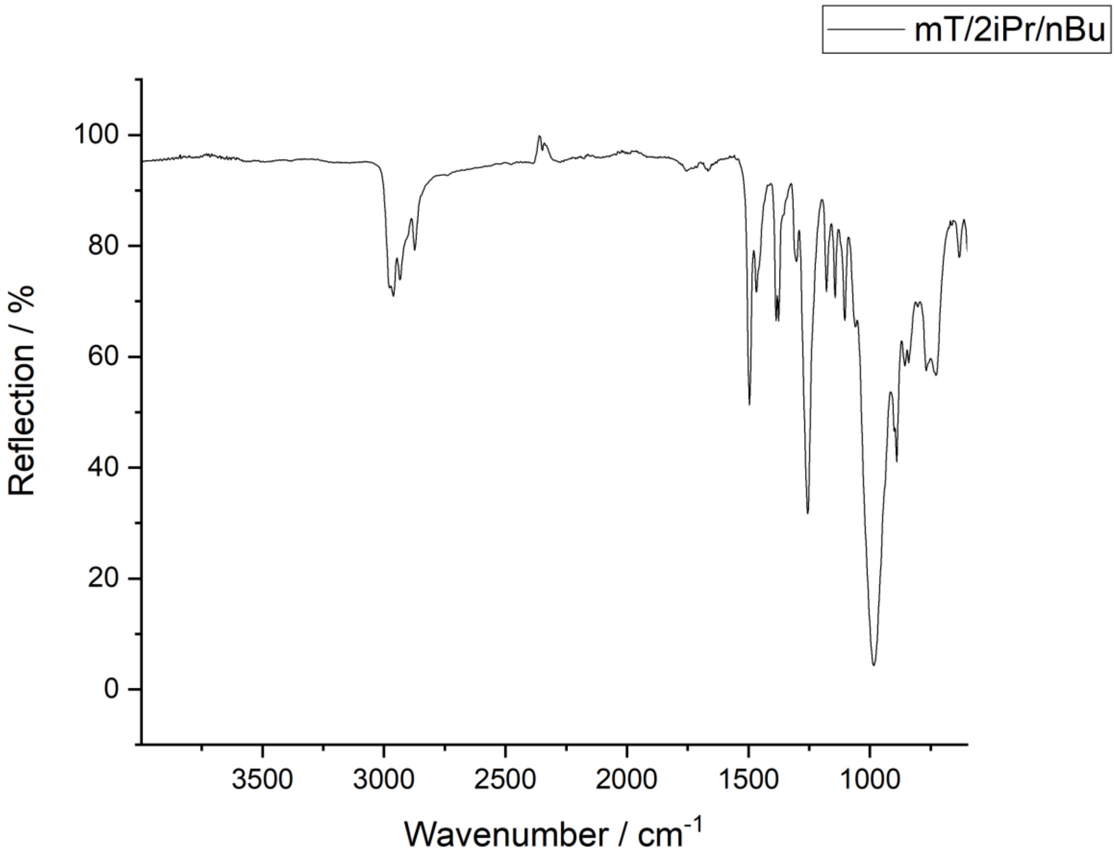

Fig. S21 ATR-IR spectrum of the mixture mT/Et/iPr/nBu

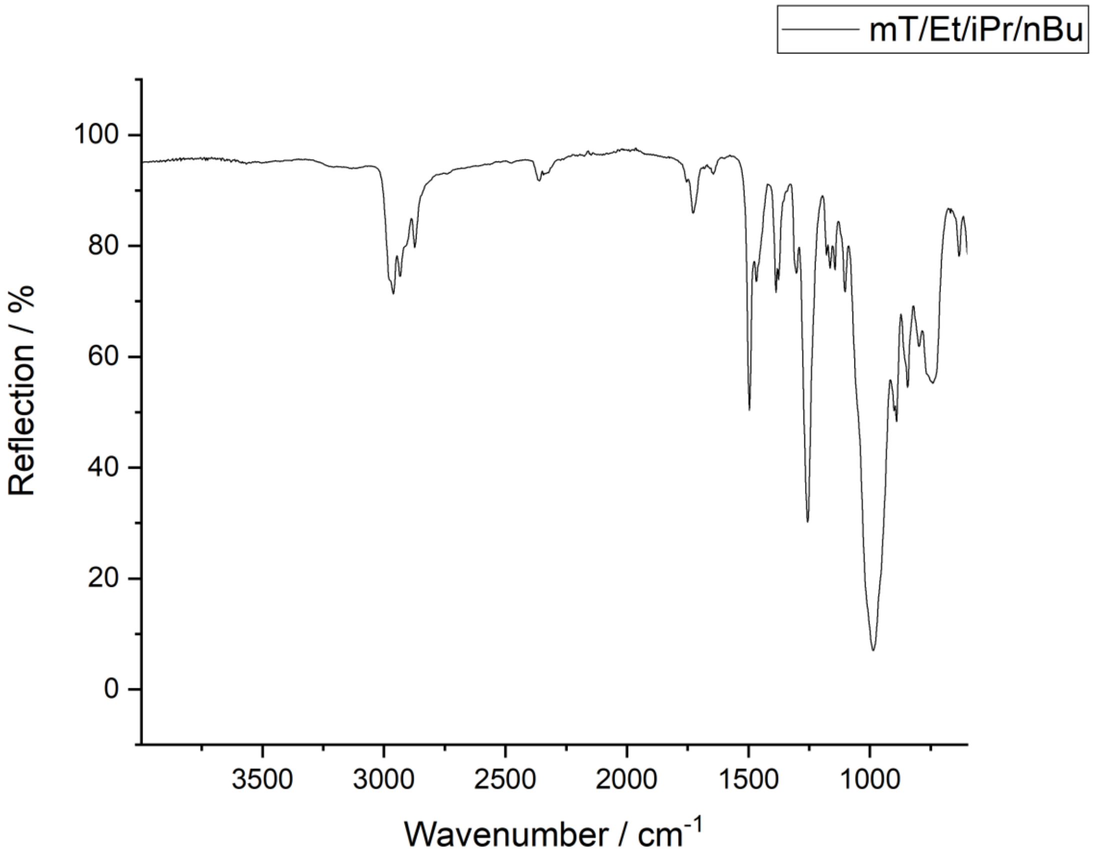

Fig. S22 ATR-IR spectrum of the mixture mT/Me/Et/iPr

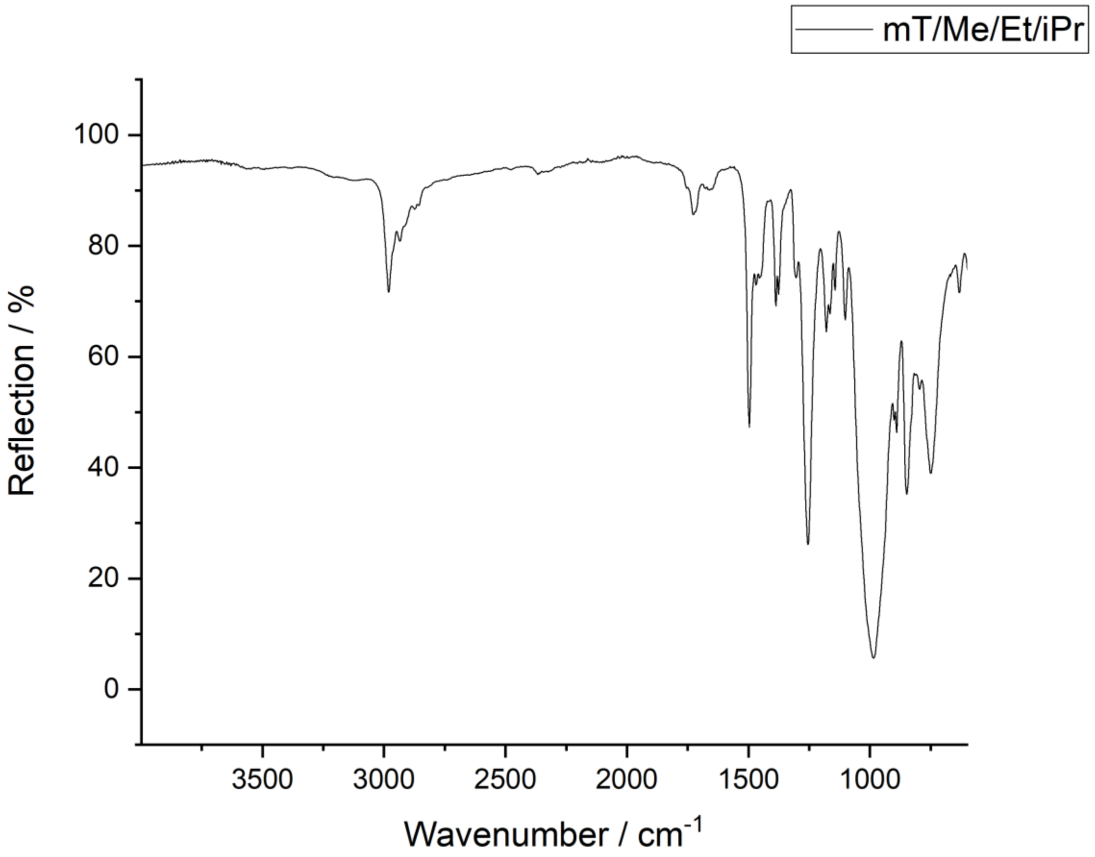

Fig. S23 ATR-IR spectrum of the mixture mT/Me/iPr/nBu

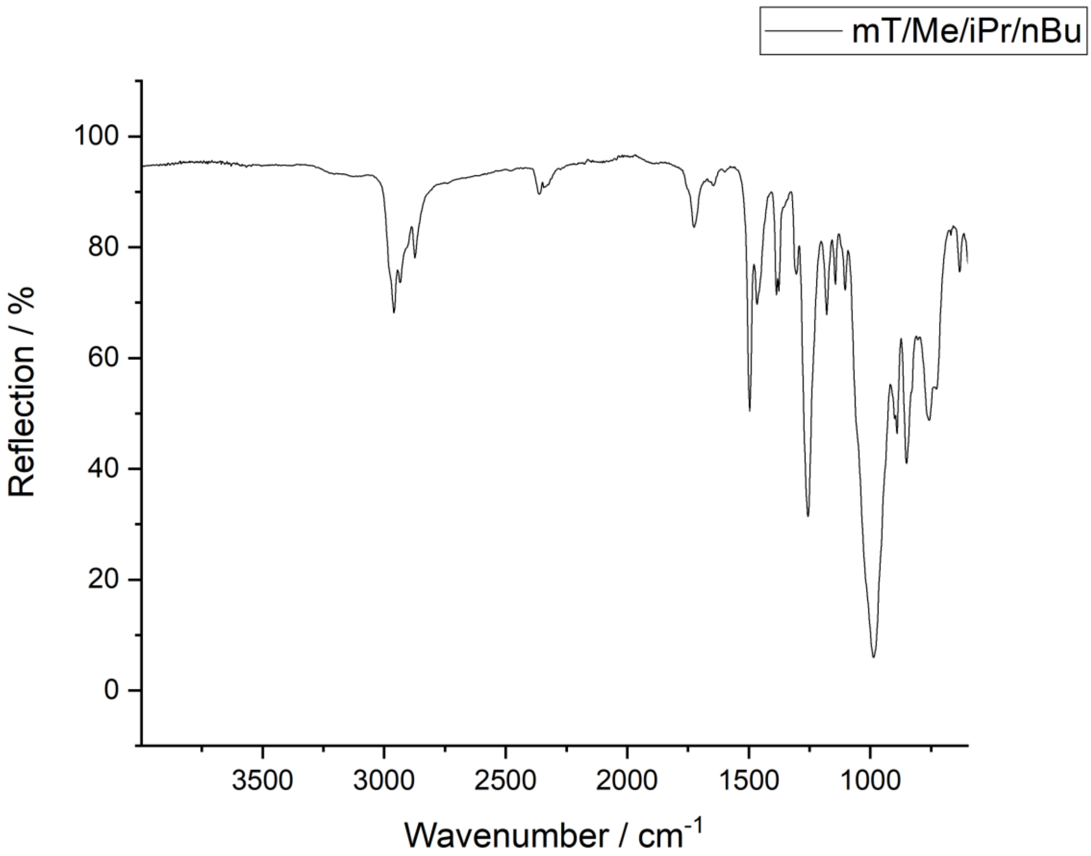

Supplement: Supplementary file 1 — Supporting Information [file OPEN-12-e202300075-s001.pdf]
